# Polycomb-lamina antagonism partitions heterochromatin at the nuclear periphery

**DOI:** 10.1038/s41467-022-31857-5

**Published:** 2022-07-20

**Authors:** Allison P. Siegenfeld, Shelby A. Roseman, Heejin Roh, Nicholas Z. Lue, Corin C. Wagen, Eric Zhou, Sarah E. Johnstone, Martin J. Aryee, Brian B. Liau

**Affiliations:** 1 Department of Chemistry and Chemical Biology, Harvard University, Cambridge, MA 02138 USA; 2 Broad Institute of Harvard and MIT, Cambridge, MA 02142 USA; 3 Department of Pathology, Dana-Farber Cancer Institute, Boston, MA 02215 USA; 4 Department of Data Science, Dana-Farber Cancer Institute, Boston, MA 02215 USA

**Keywords:** Epigenomics, Genomic analysis, Nuclear envelope

## Abstract

The genome can be divided into two spatially segregated compartments, A and B, which partition active and inactive chromatin states. While constitutive heterochromatin is predominantly located within the B compartment near the nuclear lamina, facultative heterochromatin marked by H3K27me3 spans both compartments. How epigenetic modifications, compartmentalization, and lamina association collectively maintain heterochromatin architecture remains unclear. Here we develop Lamina-Inducible Methylation and Hi-C (LIMe-Hi-C) to jointly measure chromosome conformation, DNA methylation, and lamina positioning. Through LIMe-Hi-C, we identify topologically distinct sub-compartments with high levels of H3K27me3 and differing degrees of lamina association. Inhibition of Polycomb repressive complex 2 (PRC2) reveals that H3K27me3 is essential for sub-compartment segregation. Unexpectedly, PRC2 inhibition promotes lamina association and constitutive heterochromatin spreading into H3K27me3-marked B sub-compartment regions. Consistent with this repositioning, genes originally marked with H3K27me3 in the B compartment, but not the A compartment, remain largely repressed, suggesting that constitutive heterochromatin spreading can compensate for H3K27me3 loss at a transcriptional level. These findings demonstrate that Polycomb sub-compartments and their antagonism with lamina association are fundamental features of genome structure. More broadly, by jointly measuring nuclear position and Hi-C contacts, our study demonstrates how compartmentalization and lamina association represent distinct but interdependent modes of heterochromatin regulation.

## Abstract (web summary)

Here the authors developed ‘Lamina-Inducible Methylation and Hi-C’ (LIMe-Hi-C) to simultaneously measure chromosome conformation, DNA methylation, and nuclear lamina positioning. Application of the method revealed dynamic changes upon PRC2 inhibition and an essential function of H3K27me3 in regulating sub-compartments and lamina association.

## Introduction

The spatial organization of DNA within the nucleus is critically involved in fundamental cellular processes ranging from transcription to DNA repair and replication^1–3^. Coordinated changes in DNA conformation and positioning are a hallmark of development, and alterations to proper genome organization have been directly linked to human disease states^4^. The development of pioneering imaging and molecular biology approaches has catalyzed a deeper understanding of genome structure and function^5–12^. In particular, high-throughput chromosome conformation capture (Hi-C) has revealed the fundamental role that A/B compartmentalization plays in overall 3D genome architecture. A/B compartmentalization is thought to be driven by association with different nuclear landmarks as well as multivalent interactions specified by chromatin context and transcriptional activity^13–16^. Specifically, the B compartment is highly correlated with chromatin that associates with the nuclear lamina, a meshwork of intermediate filament proteins at the inner membrane of the nuclear envelope^1,11,17^. Lamina-associated domains (LADs) constitute nearly 40% of the mammalian genome and are characterized by large blocks of CpG hypomethylation and enrichment in repressive histone modifications, including H3K9me2/3 and H3K27me3^10,17–25^. H3K9me2/3 and H3K27me3 are segregated into distinct classes of LADs, consistent with their divergent roles in mediating constitutive and facultative heterochromatin, respectively^26–28^. However, H3K27me3 is also highly enriched at the borders of compartments and LADs, raising questions about the precise role of this mark in regulating lamina association^10,17,23,24^.

Beyond its presence within LADs, H3K27me3 has important roles in the cell type-specific regulation of gene expression and genome organization across both A and B compartments^1,26,29^. Recent studies have demonstrated that high levels of H3K27me3, often within CpG hypomethylated regions, can mediate long-range chromatin interactions spanning from DNA loops to self-associating domains^30–37^. These H3K27me3-mediated interactions have been implicated in the regulation of key developmental genes and are distinct from canonical structures regulated by CTCF and cohesin^30–37^. However, how Polycomb interactions and H3K27me3 impact compartmentalization, nuclear positioning, and heterochromatin organization remains to be fully resolved^21,38^. Deconvoluting how these layers of genome organization control cell-type-specific expression programs will further our understanding of Polycomb Group (PcG) proteins and the roles they play in development and disease^39^.

In this work, we develop Lamina-Inducible Methylation and Hi-C (LIMe-Hi-C) to simultaneously measure lamina association, DNA methylation and chromatin contacts. Through this approach, we identify sub-compartments with variable levels of DNA methylation and lamina association with distinct epigenomic landscapes. We subsequently apply LIMe-Hi-C following inhibition of PRC2 and DNA methyltransferase 1 (DNMT1) to study the influence of these key epigenomic regulators on overall chromatin structure. While DNMT1 inhibition leads to larger changes in compartmentalization, PRC2 inhibition leads to more extensive changes in lamina association, suggesting that these interconnected architectural features are distinctly regulated. Unexpectedly, H3K27me3-marked B compartment regions at LAD and compartment borders move to the lamina following PRC2 inhibition but not DNMT1 inhibition. This relocalization is accompanied by H3K9me3 spreading and sustained gene repression suggesting that PRC2 antagonizes lamina association and buffers constitutive heterochromatin from facultative heterochromatin. These results provide insights regarding the influence of PRC2 on genome organization and the role of H3K27me3 at the boundaries of LADs and compartments.

## Results

### LIMe-Hi-C simultaneously identifies LADs, CpG methylation, and Hi-C contacts

**Fig. 1:**
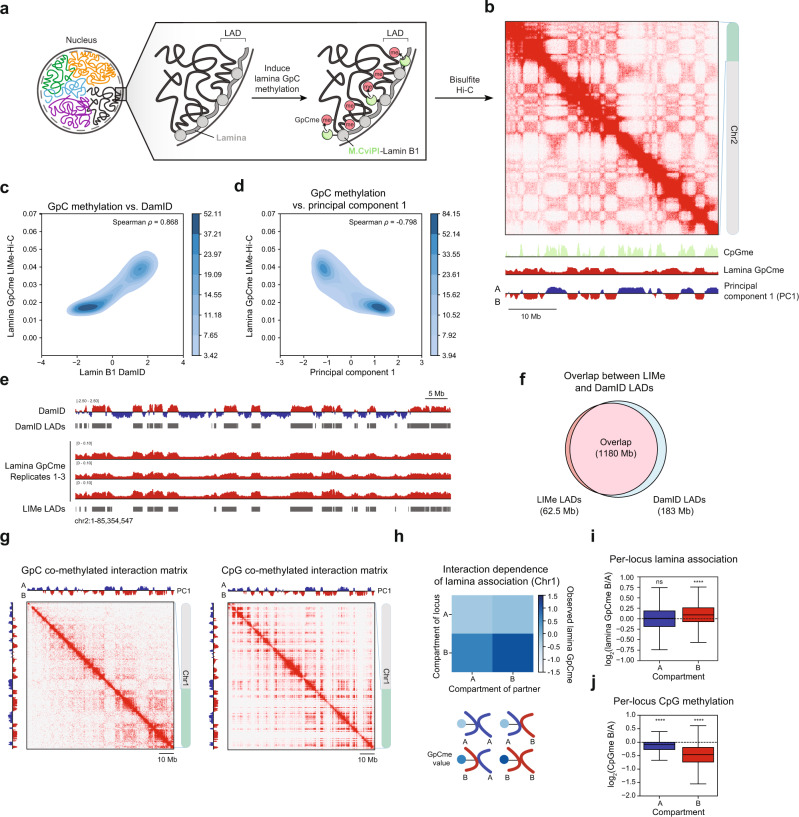
LIMe-Hi-C simultaneously identifies LADs, CpG methylation, and Hi-C contacts. **a** Schematic of the LIMe-Hi-C workflow. **b** LIMe-Hi-C contact map for replicate 1 with lamina GpC methylation, CpG methylation, and principal component 1 at 100 kb resolution. **c** Density heatmap comparing DamID signal (*x*-axis, Dam-Lamin B1/Dam-only enrichment) to replicate-averaged LIMe-Hi-C GpC methylation fraction (*y*-axis) across 50 kb bins. Published datasets are specified in Supplementary Table 1. **d** Density heatmap comparing replicate-averaged principal component 1 (*x*-axis) to replicate-averaged LIMe-Hi-C GpC methylation fraction (*y*-axis) across 50 kb bins. **e** Genome browser tracks depicting lamina GpC methylation signal, LIMe LADs, DamID signal, and DamID LADs. Published datasets are specified in Supplementary Table 1. **f** Venn diagram describing the genome-wide base pair overlap between LIMe LADs and published DamID LADs. **g** Example Hi-C interaction matrix merged across replicates for a region on chromosome 1 depicting only contacts where both reads are GpC methylated (left) or CpG methylated (right). **h** Normalized average lamina GpC methylation for loci as a function of the compartment status of the loci’s interaction partner for chromosome 1 (see Methods). Curved lines represent the compartment identities of the DNA regions within each interaction pair. **i** Boxplot across 50 kb bins genome-wide for both compartments (*x*-axis) depicting the log_2_ ratio of the interval’s lamina association status (*y*-axis) if it is interacting with the B versus the A compartment. *P* values were calculated by a one-sample one-sided t-test to determine if the mean is greater than 0. **j** Boxplot across 50 kb bins genome-wide for both compartments (*x*-axis) depicting the log_2_ ratio of the interval’s CpG methylation status (*y*-axis) if it is interacting with the B versus the A compartment. *P* values were calculated by a one-sample one-sided t-test to determine if the mean is less than 0. In (**i**, **j**) the interquartile range (IQR) is depicted by the box with the median represented by the center line. Whiskers maximally extend to 1.5 × IQR (with outliers excluded). *P* values are annotated as follows: ns: not significant; *: 0.01 < *p* ≤ 0.05; **: 0.001 < *p* ≤ 0.01; ***: 0.0001 < *p* ≤ 0.001; ****: *p* ≤ 0.0001. Exact *p* values and the number of datapoints (*n*) compared are provided in the source data file.

Motivated by the strong connection between nuclear localization and DNA topology, we developed an approach, Lamina-Inducible Methylation and Hi-C (LIMe-Hi-C), that combines the detection of protein-DNA interactions at the nuclear lamina with Hi-C to superimpose spatial information on DNA interaction data. Recent methods integrating bisulfite conversion into the Hi-C workflow have enabled the simultaneous detection of endogenous CpG DNA methylation and Hi-C contacts, suggesting that tracking lamina association through introducing exogenous cytosine methylation could be achieved^40–42^. We fused the GpC cytosine methyltransferase, M.CviPI, to Lamin B1, in a strategy analogous to DNA adenine methyltransferase identification (DamID) (Fig. 1a)^10,43,44^. Detection of this GpC methylation signature can be directly integrated into a bisulfite Hi-C workflow to simultaneously map chromatin structure, lamina association, and native CpG methylation (see Methods).

To perform LIMe-Hi-C, we first generated a clonal K562 cell line that expresses the Flag-M.CviPI-Lamin B1 fusion protein upon addition of doxycycline (dox). Using this cell line, LIMe-Hi-C was performed in triplicate after 24 hours of dox treatment through leveraging the bisulfite Hi-C method, Hi-Culfite^40^, to generate Hi-C contacts with superimposed information mapping endogenous CpG methylation and exogenous GpC methylation indicative of lamina association (Fig. 1b, Supplementary Fig. 1a). The bisulfite Hi-C contact data and CpG methylation profile obtained from LIMe-Hi-C are highly similar to those we obtained from performing standard in situ Hi-C and analyzing published whole-genome bisulfite sequencing data for K562, respectively (Supplementary Fig. 1b, c)^11,40^. In addition, all three biological replicates exhibited similar profiles of GpC methylation and principal component 1 (PC1), the first eigenvector of the Hi-C Pearson correlation matrix, confirming that LIMe-Hi-C provides robust measurements of Hi-C contacts and lamina association (Spearman *ρ* > 0.95) (Supplementary Fig. 1d, e).

Consistent with the capacity of LIMe-Hi-C to profile lamina association, as well as the reported connection between the B compartment and LADs, the lamina GpC methylation signal was strongly correlated with the Lamin B1 DamID signal and anticorrelated with PC1, a measure of A/B compartmentalization (Fig. 1b–d). By contrast, in the absence of induction with dox, virtually no lamina GpC methylation signal was observed (Supplementary Fig. 1f). In addition, both the Hi-C contact heatmaps and PC1 scores are highly concordant irrespective of dox treatment, suggesting that expression of the fusion did not significantly perturb genome structure (Supplementary Fig. 1f, g). Next, we called LADs from the LIMe-Hi-C GpC methylation data after normalizing the GpC methylation signal^45,46^. We identified 1,165 LADs with a median size of 600 kb, which significantly overlap the 1,247 LADs of median size 580 kb called by DamID (Fig. 1e, f, Supplementary Fig. 1h) (Supplementary Data 1). Taken together, these results show that combining inducible expression of M.CviPI fused to Lamin B1 with bisulfite Hi-C enabled the robust identification of LADs, CpG methylation, and DNA-DNA contacts in a single experimental workflow.

Because LIMe-Hi-C provides single-molecule readouts of lamina association, Hi-C contact frequencies, and CpG methylation, we next investigated whether it could reveal correlations between epigenomic features that are otherwise obscured in population average measurements. Consistent with previous findings^40^, Hi-C contact pairs on chromosome 1 are more likely than random chance to have the same CpG methylation status, suggesting a high degree of endogenous DNA methylation coordination (Supplementary Fig. 1i). To study lamina association through an analogous approach, we analyzed per-read GpC methylation data for chromosome 1 and observed a strong enrichment in contacts sharing the same lamina association status (Supplementary Fig. 1i), supporting prior models in which lamina association within single cells is correlated with bulk Hi-C interaction measurments^17^. Taking advantage of the multi-modal data, we isolated Hi-C interaction matrices specifically containing contacts where both interaction partners possess lamina association or CpG methylation signal. The lamina-associated matrix for chromosome 1 exhibits strong enrichment of B-B interactions whereas the CpG-methylated version exhibits strong enrichment of A-A interactions (Fig. 1g). We also observed that B compartment regions display higher average lamina association signal when interacting with other partners from the B versus the A compartment (Fig. 1h), suggesting that a region’s interaction partner impacts its lamina association frequency. In addition, for every 50 kb region genome-wide, we examined the ratio of observed lamina association signals when the same region interacted with the B versus the A compartment, revealing that B compartment regions were more likely to be lamina associated when in contact with other B compartment regions (Fig. 1i). The opposite trend is observed for CpG methylation, where B compartment loci are more likely to be CpG methylated when interacting with A compartment regions, consistent with the A compartment possessing higher levels of endogenous CpG methylation in K562 cells (Fig. 1j)^33^. These data illustrate how the nuclear localization preferences and endogenous CpG methylation status of the same genomic region can vary depending on the types of loci the region is contacting. Altogether, we demonstrate that LIMe-Hi-C provides multi-modal information that can illuminate the interplay between lamina association and DNA conformation.

### Identification of topologically distinct Polycomb sub-compartments with LIMe-Hi-C

**Fig. 2:**
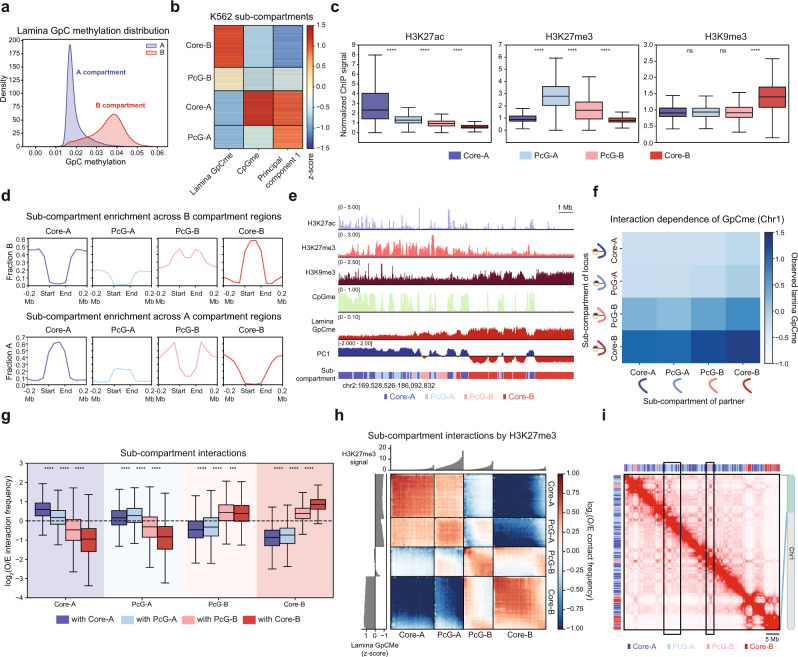
Identification of topologically distinct Polycomb sub-compartments with LIMe-Hi-C. **a** Density plot depicting the relative density (*y*-axis) of the average fraction lamina GpC methylation (*x*-axis) for 50 kb bins across compartments. The density function for each compartment is independently scaled. **b** Heatmap of 50 kb genomic regions clustered into sub-compartments identified by *k*-means clustering. Color represents the *z*-score-transformed value for LIMe features across every genomic bin. **c** Boxplot showing levels of histone modifications (*y*-axis, fold-change over input/median signal) for 50 kb bins across sub-compartments (*x*-axis). Published datasets are specified in Supplementary Table 1. **d** Aggregate plot depicting the fraction of a specified sub-compartment type (*y*-axis) across compartment regions (*x*-axis) for the B compartment (top) and A compartment (bottom). **e** Representative genome browser tracks depicting histone ChIP-seq signal along with CpG methylation (replicate 1), lamina GpC methylation (replicate 1), and principal component 1 (replicate 1). The sub-compartment designation is included below. Published datasets are specified in Supplementary Table 1. **f** Normalized average lamina GpC methylation for loci as a function of the sub-compartment status of the loci’s interaction partner for chromosome 1 (see Methods). Curved lines represent the sub-compartment identities of the DNA regions within each interaction pair. **g** Boxplot showing average log_2_(observed/expected contact frequency) for 50 kb bin level interactions (*y*-axis) across sub-compartments (*x*-axis). O/E denotes observed/expected. **h** Summary interaction heatmap ordered by sub-compartment and H3K27me3 ChIP-seq signal of the log_2_(observed/expected contact frequency). Replicate-averaged *z*-score lamina GpC methylation (left) and H3K27me3 levels (top) per quantile are displayed alongside the interaction heatmap. O/E denotes observed/expected. **i** LIMe-Hi-C contact map merged across the three replicates. Example regions are highlighted with black boxes. In (**c**, **g**) the interquartile range (IQR) is depicted by the box with the median represented by the center line. Whiskers maximally extend to 1.5 × IQR (with outliers excluded). *P* values were calculated by a Mann-Whitney-Wilcoxon two-sided test and are annotated as follows: ns: not significant; *: 0.01 <*p* ≤ 0.05; **: 0.001 <*p* ≤ 0.01; ***: 0.0001 <*p* ≤ 0.001; ****: *p* ≤ 0.0001. Exact *p* values and the number of datapoints (*n*) compared are provided in the source data file.

Although lamina association is generally correlated with the B compartment, we observed a large degree of variability in the lamina GpC methylation signal—even for regions in the B compartment with similar PC1 values (Fig. 1d, Fig. 2a, Supplementary Fig. 2a). In addition, in K562 cells, the B compartment is depleted of CpG methylation while the A compartment possesses both hypomethylated and hypermethylated regions (Supplementary Fig. 2a, b). These observations prompted us to classify the genome into regions based on CpG methylation, lamina association, and PC1 scores. *K*-means clustering identified sub-compartment types characterized by their variance across these features (Fig. 2b, Supplementary Fig. 2c) (Supplementary Data 2). We termed these sub-compartments Core-A, PcG-A, PcG-B, and Core-B. As described below, the Core-A and Core-B sub-compartments possess the canonical features typically associated with the A and B compartments, respectively. By contrast, the PcG-A and PcG-B sub-compartments are both enriched for the PRC2 mark, H3K27me3, and are consequently given the Polycomb group (PcG) designation.

Using this classification system, A compartment regions are largely differentiated by their CpG methylation status (Fig. 2b, Supplementary Fig. 2c). CpG-hypermethylated Core-A regions are highly enriched in H3K27ac while hypomethylated PcG-A regions are enriched in H3K27me3 (Fig. 2c). By contrast, B compartment regions are chiefly differentiated by their variable association with the nuclear lamina. Core-B regions with the highest levels of lamina association are enriched in H3K9me3 (Fig. 2c) and are located within the interior of the B compartment (Fig. 2d, e). By contrast, PcG-B regions have PC1 scores closer to zero (less B-like), lower levels of lamina association, and are enriched in H3K27me3 (Fig. 2c, Supplementary Fig. 2c). Consistent with the enrichment of H3K27me3 within PcG-A and PcG-B, these sub-compartments also display moderate enrichment of proteins associated with PRC1 and PRC2 (Supplementary Fig. 2d) and overlap H3K27me3 peaks to the greatest extent in comparison to Core-A and Core-B (Supplementary Fig. 2e, f). Furthermore, B compartment regions that overlap H3K27me3 peaks are less lamina associated than B compartment regions that do not (Supplementary Fig. 2g). PcG-B regions are often located at compartment borders, suggesting that these regions could be involved in boundary formation (Fig. 2d).

We next investigated the relationship between the sub-compartments we identified and LAD sub-types previously called in KBM7 cells. Despite being different cell lines, KBM7 and K562 have been compared previously^17^. As expected, Core-B is enriched in constitutive LADs, which are lamina associated across a variety of cell types, whereas Core-A is enriched in constitutive inter-LADs, which never associate with the lamina (Supplementary Fig. 2h). By contrast, PcG-B and PcG-A are comprised predominantly of facultative LADs and facultative inter-LADs, which exhibit variable association with the nuclear lamina across cell types, consistent with the role of H3K27me3 in mediating cell-type-specific gene expression programs (Supplementary Fig. 2h). Lastly, we examined the lamina association of Hi-C interaction pairs at a per-contact level. We found that the lamina association frequency of a given PcG-B locus is strongly influenced by its contact partner for regions on chromosome 1 (Fig. 2f). In particular, B sub-compartment regions are less prone to be lamina associated when interacting with PcG-B regions compared to Core-B regions, supporting the intermediate positioning of PcG-B relative to the nuclear periphery (Supplementary Fig. 2i-j).

As H3K27me3 has been implicated in mediating chromatin interactions^30–37^, we next considered if PcG-A and PcG-B regions (1) exhibit a preference for self-interactions and (2) are topologically distinct from their parental compartments. Indeed, PcG-A, PcG-B, Core-A, and Core-B regions exhibit stronger self-association within their own respective regions. Specifically, the average per-bin observed/expected Hi-C interaction frequency within a classified region is typically greater than its interaction frequency with other regions (Fig. 2g, h). This behavior is consistent when considering replicates separately and when averaging interactions across the genome (Supplementary Fig. 2k). Although PcG-B has a weaker propensity to self-interact, Core-B strongly self-associates (Fig. 2g, h), suggesting that Core-B and PcG-B also segregate separately. Moreover, the interaction preferences of PcG-B regions vary depending on their baseline levels of H3K27me3. Specifically, PcG-B domains with the highest levels of H3K27me3 exhibit interaction patterns more similar to regions within PcG-A, while PcG-B domains with the lowest levels of H3K27me3 exhibit interaction patterns more similar to regions within Core-B (Fig. 2h). Lastly, although largely partitioned into opposing compartments, PcG-A and PcG-B display moderate levels of cross-compartment Hi-C interactions, which are also observable on Hi-C contact matrices (Fig. 2i). These data suggest that H3K27me3 marks a class of repressed chromatin embedded within the otherwise active A compartment and partitions the B compartment into two distinct sub-compartments that maintain differential contacts with the nuclear lamina and the Polycomb-repressed A compartment.

### Inhibition of EZH2 and DNMT1 differentially remodel chromatin compartmentalization

To investigate if H3K27me3 controls the structure and localization of Polycomb sub-compartments and the genome at large, we conducted LIMe-Hi-C in triplicate after chemical inhibition of EZH2, the core catalytic subunit of PRC2, with GSK343 (EZH2i)^47^. To provide a comparator to EZH2 inhibition and benchmark the robustness of the technique, we also performed LIMe-Hi-C following treatment with the non-covalent DNA methyltransferase 1 (DNMT1) inhibitor, GSK3482364 (DNMT1i)^48,49^. The inhibitors were dosed for 3 days at 1 µM and 10 µM, respectively, which led to negligible growth defects (Supplementary Fig. 3a). We verified that levels of the Flag-M.CviPI-Lamin B1 fusion were comparable across drug treatments (Supplementary Fig. 3b). Quantitative spike-in ChIP-seq demonstrated that treatment with EZH2i depleted H3K27me3 genome-wide (Supplementary Fig. 3c, d). The magnitude of a region’s change in H3K27me3 was correlated with its baseline level of H3K27me3 (Supplementary Fig. 3c), and PcG-A and PcG-B exhibited the greatest decreases in H3K27me3, consistent with these sub-compartments possessing the highest baseline levels of this modification (Fig. 2c, Supplementary Fig. 3e). As detected through LIMe-Hi-C, treatment with DNMT1i led to an approximately 4-fold reduction in endogenous CpG DNA methylation signal, with Core-A (Supplementary Fig. 3f, g), the only highly methylated sub-compartment, losing the greatest amount of DNA methylation (Supplementary Fig. 3h).

**Fig. 3:**
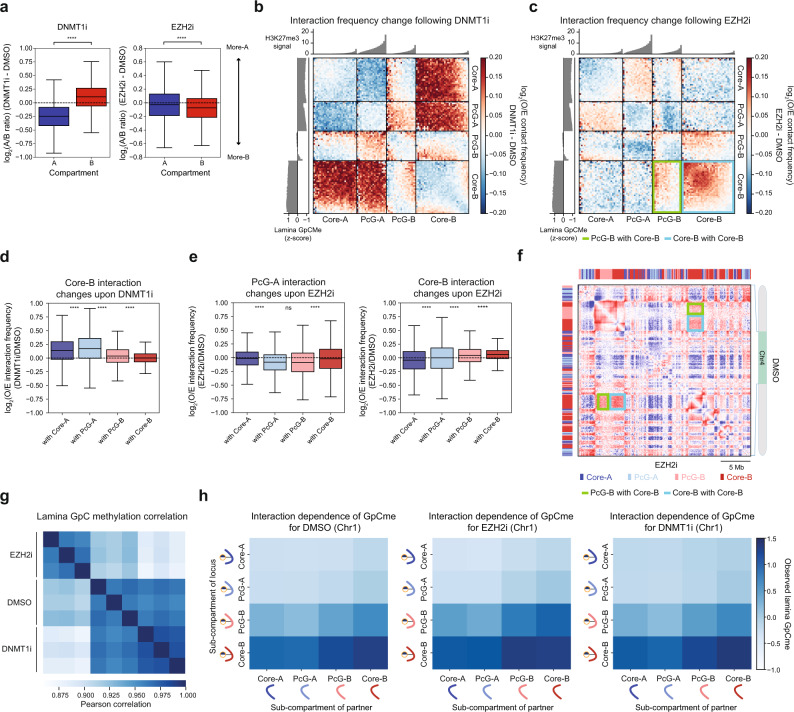
Inhibition of EZH2 and DNMT1 differentially remodel chromatin compartmentalization. **a** Boxplot depicting replicate-averaged difference in log_2_(A/B ratio) for 50 kb bins (*y*-axis) (see Methods) between inhibitor treatment and vehicle treatment across compartments (*x*-axis). **b** Summary interaction heatmap ordered by sub-compartment and baseline H3K27me3 ChIP-seq signal of the difference in the log_2_(observed/expected contact frequency) averaged across replicates between DNMT1 inhibition and vehicle treatment. Averaged baseline *z*-score lamina GpC methylation (left) and H3K27me3 (top) levels per quantile are displayed alongside the interaction heatmap. **c** Summary interaction heatmap ordered by sub-compartment and baseline H3K27me3 ChIP-seq signal of the difference in the log_2_(observed/expected contact frequency) averaged across replicates between EZH2 inhibition and vehicle treatment. Averaged baseline *z*-score lamina GpC methylation (left) and H3K27me3 (top) levels per quantile are displayed alongside the interaction heatmap. Example interaction changes are highlighted with colored boxes. **d** Boxplot showing replicate-averaged difference in log_2_(observed/expected contact frequency) of every 50 kb bin (*y*-axis) for DNMT1 inhibition relative to vehicle treatment for interactions with Core-B across sub-compartments (*x*-axis). **e** Boxplot showing replicate-averaged difference in log_2_(observed/expected contact frequency) of every 50 kb bin (*y*-axis) for EZH2 inhibition relative to vehicle treatment for interactions with PcG-A (left) and Core-B (right) across sub-compartments (*x*-axis). **f** Representative Hi-C observed/expected contact map comparing EZH2 inhibitor to vehicle treatment. Contacts from the three replicates were pooled together. Example interaction changes are highlighted with colored boxes. **g** Pearson correlation heatmap of *z*-score-normalized GpC methylation across 50 kb bins for LIMe-Hi-C samples. **h** Normalized average lamina GpC methylation for loci as a function of the sub-compartment status of the loci’s interaction partner for vehicle and inhibitor treatments for chromosome 1 (see Methods). Curved lines represent the sub-compartment identities of the DNA regions within each interaction pair. In (**a**, **d**, **e**) the interquartile range (IQR) is depicted by the box with the median represented by the center line. Whiskers maximally extend to 1.5 × IQR (with outliers excluded). *P* values were calculated by a Mann-Whitney-Wilcoxon two-sided test and are annotated as follows: ns: not significant; *: 0.01 < *p* ≤ 0.05; **: 0.001 < *p* ≤ 0.01; ***: 0.0001 < *p* ≤ 0.001; ****: *p* ≤ 0.0001. Exact *p* values and the number of datapoints (*n*) compared are provided in the source data file. O/E denotes observed/expected.

At a global level, EZH2 and DNMT1 inhibition led to largely contrasting changes in compartmentalization with DNMT1 inhibition impacting compartmentalization to a larger degree than EZH2 inhibition (Fig. 3a–c, Supplementary Fig. 3i). In agreement with prior studies, DNMT1 inhibition reduced genome compartmentalization on a global scale^34,50,51^, which is reflected by the decreased ratio of intra- to inter-compartment interactions as well as broad compartment shifts, with B regions becoming more A-like and vice versa (Fig. 3a). At the sub-compartment level, these shifts included strengthened interactions between Core-B and both A sub-compartments (Fig. 3b, d). By contrast, EZH2 inhibition did not globally alter compartmentalization to as great an extent, but instead led to a modest strengthening of B-compartment interactions (Fig. 3a). These effects were unexpected since Polycomb mediates chromatin compaction and has been hypothesized to be implicated in lamina association^10,16,24,52^. To better understand the effects of EZH2 inhibition on compartmentalization, we assessed sub-compartment-specific changes in genome organization. Sub-compartment-specific analysis revealed more granular changes in interactions within the A compartment that were obscured when PcG-A and Core-A were grouped together (Fig. 3c). Specifically, EZH2 inhibition generally decreased Hi-C self-interactions within PcG-A, which contains the highest levels of H3K27me3 (Fig. 3c, e). These data support the notion that H3K27me3 mediates chromatin interactions that contribute to the segregation of sub-compartments within the larger A compartment.

We next scrutinized the effects of EZH2 inhibition on the B sub-compartments. Upon EZH2 inhibition, PcG-B regions gained modest interactions with Core-B regions, which exhibited a concomitant increase in Hi-C self-interactions that drive enhanced B compartmentalization (Fig. 3e, f). This increase in Core-B interactions suggested that H3K27me3 may be acting as a barrier to B compartment interactions. Consistent with this idea, EZH2 inhibition decreased interactions between PcG-B and PcG-A (Fig. 3c, e), suggesting that H3K27me3 may support interactions between these sub-compartments. Notably, PcG-B is heterogeneous, with regions possessing the highest levels of H3K27me3 gaining fewer B compartment interactions upon EZH2 inhibition (Fig. 3c); it is possible that such regions may share more similarities with PcG-A. Minimal changes in CpG methylation were observed following EZH2 inhibition, indicating that alterations in DNA methylation were not driving compartment changes (Supplementary Fig. 3j, k). Taken together, these findings suggest that H3K27me3 establishes topologically distinct sub-compartments in part by promoting interactions within PcG-A and PcG-B sub-compartments while acting as an unexpected barrier to overall B compartmentalization.

Given these observed changes in compartmentalization, we next assessed whether lamina association was substantially altered following either EZH2 or DNMT1 inhibition. Surprisingly, despite the broader compartment shifts observed upon DNMT1 inhibition (Fig. 3a, Supplementary Fig. 3i), lamina association signal at a global scale was altered to a greater extent upon EZH2 inhibition (Fig. 3g). LIMe-Hi-C per-read analysis was consistent with the more pronounced changes in lamina contacts observed upon EZH2 inhibition relative to DNMT1 inhibition. Specifically, upon EZH2 inhibitor treatment, Core-B showed higher levels of lamina association relative to vehicle treatment when interacting with the PcG-B sub-compartment, suggesting facultative heterochromatin may be repositioning from the interior to the nuclear periphery (Fig. 3h, Supplementary Fig. 3l). Altogether, these results for EZH2 and DNMT1 inhibition demonstrate that large-scale changes in lamina association are not necessarily directly coupled with changes in compartmentalization.

### PRC2 antagonizes lamina association and constitutive heterochromatin spreading

**Fig. 4:**
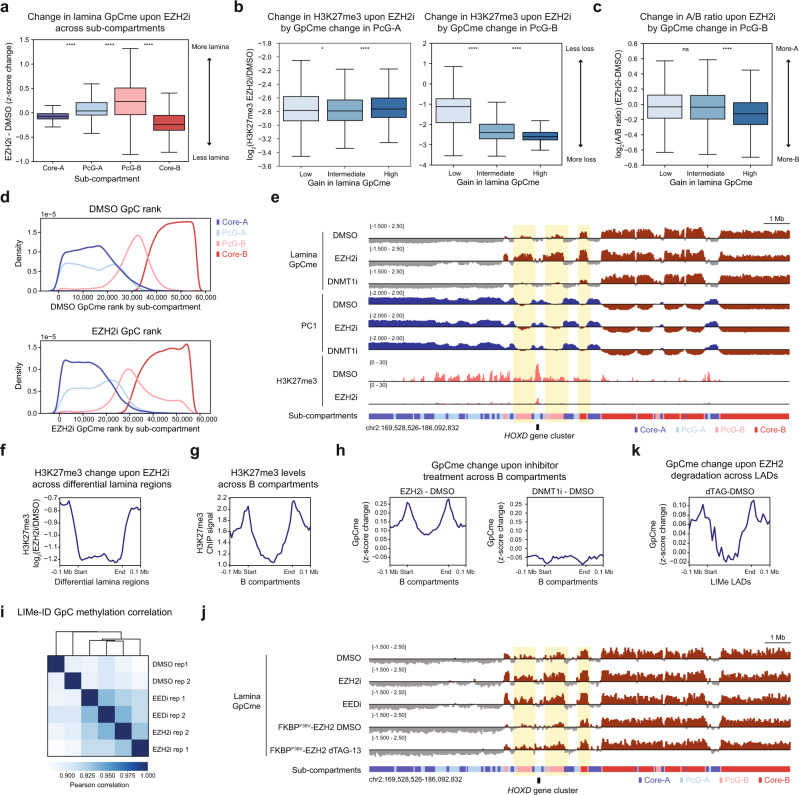
PRC2 antagonizes lamina association. **a** Boxplot showing *z*-score-normalized GpC methylation change between EZH2i and vehicle treatment for 50 kb bins (*y*-axis) across sub-compartments (*x*-axis). **b** Boxplot showing log_2_ fold-change in H3K27me3 levels between EZH2i and vehicle treatment for 50 kb bins (*y*-axis) segregated into three equally sized quantiles by change in *z*-score-normalized lamina GpC methylation (*x*-axis) for PcG-A (left) and PcG-B (right). **c** Boxplot showing change in log_2_(A/B ratio) between EZH2i and vehicle treatment for 50 kb bins (*y*-axis) segregated into three equally sized quantiles by change in *z*-score-normalized GpC methylation (*x*-axis) for PcG-B. **d** Density plot depicting density (*y*-axis) of average lamina GpC methylation bin rank value across replicates (*x*-axis) for vehicle treatment (top) and EZH2 inhibitor treatment (bottom) for 50 kb bins colored by sub-compartment designation. **e** Genome browser tracks of replicate-averaged *z*-score-normalized lamina GpC methylation levels, principal component 1, and H3K27me3 ChIP-seq signal for LIMe-Hi-C and ChIP-seq data near the *HOXD* locus. **f** Aggregate profile plot of log_2_ fold-change H3K27me3 ChIP-seq signal between EZH2 inhibition and vehicle treatment (*y*-axis) across regions gaining lamina contact upon EZH2 inhibition (*x*-axis) (see Methods). **g** Aggregate profile plot of baseline H3K27me3 ChIP-seq signal for vehicle treatment (*y*-axis) across B compartments (*x*-axis). **h** Aggregate profile plots depicting change in *z*-score-normalized lamina GpC methylation (*y*-axis) across B compartment regions (*x*-axis) upon inhibitor treatments. **i** Clustered Pearson correlation heatmap of *z*-score-normalized GpC methylation across 250 kb bins for LIMe-ID data. **j** Genome browser tracks of replicate-averaged *z*-score-normalized lamina GpC methylation levels for LIMe-ID data near the *HOXD* locus. **k** Aggregate profile plots depicting change in *z*-score-normalized lamina GpC methylation (*y*-axis) across LIMe LADs (*x*-axis) for EZH2 degradation relative to vehicle treatment. In (**a**–**c**) the interquartile range (IQR) is depicted by the box with the median represented by the center line. Whiskers maximally extend to 1.5 × IQR (with outliers excluded). *P* values were calculated by a Mann-Whitney-Wilcoxon two-sided test and are annotated as follows: ns: not significant; *: 0.01 < *p* ≤ 0.05; **: 0.001 < *p* ≤ 0.01; ***: 0.0001 < *p* ≤ 0.001; ****: *p* ≤ 0.0001. Exact *p* values and the number of datapoints (*n*) compared are provided in the source data file.

Prompted by the unanticipated observation that EZH2 inhibition strengthened B compartment interactions and altered lamina-associated contacts, we explored sub-compartment-specific changes in lamina association. Both Polycomb sub-compartments preferentially gained lamina association upon EZH2 inhibition (Fig. 4a). Regions within PcG-B that became the most lamina-attached lost the most H3K27me3 as determined by quantitative ChIP-seq (Fig. 4b). Notably, this trend is not observed for PcG-A (Fig. 4b). Furthermore, PcG-B regions that gained the most lamina association also became more B-like, although the trend is not observed for regions gaining intermediate lamina attachment (Fig. 4c). This observation is consistent with our findings that changes in compartmentalization are related to but not always directly linked to changes in lamina association when comparing the effects of the DNMT1 and EZH2 inhibitor treatments. This shifting of PcG-B to the periphery is also evidenced by examining the change in lamina GpC methylation rank between EZH2 inhibitor and vehicle treatment (Fig. 4d). By contrast, DNMT1 inhibition did not lead to an increase in lamina attachment for PcG-B regions (Supplementary Fig. 4a). Thus, despite prior models indicating that in certain contexts Polycomb may mediate lamina association and cooperate with constitutive heterochromatic factors such as HP1^1,24,53–55^, our findings suggest that H3K27me3 antagonizes direct lamina contact and interferes with B compartment interactions, adding another layer of complexity to these models. Such antagonism is exemplified near the key developmental *HOXA* and *HOXD* loci, where neighboring PcG-B domains enriched in H3K27me3 gain lamina association and, in some cases, modest B compartment interactions following EZH2 inhibition (Fig. 4e, Supplementary Fig. 4b).

To explore PRC2-lamina antagonism further, we directly called differential lamina-associated regions between EZH2 inhibitor and vehicle treatment, which led to the identification of 912 regions of at least 150 kb in size that gained lamina contact (average size 301.7 kb) (Supplementary Data 3). Utilizing the same statistical thresholds, only 236 regions (average size 176.3 kb) were identified that lost contact with the lamina. Consistent with our prior analysis (Fig. 4b–d), the regions that gained lamina association significantly overlapped PcG-B and were highly depleted of H3K27me3 by EZH2 inhibition (Fig. 4f, Supplementary Fig. 4c, d). By contrast, regions that lost lamina association were observed to predominantly overlap Core-B (Supplementary Fig. 4e) but comprised a significantly smaller proportion of the genome compared to regions that gain lamina association and may have been identified due to the z-score standardization employed.

Given that H3K27me3 is enriched at LAD and compartment borders (Fig. 4g, Supplementary Fig. 4f, g)^10,24^, we considered whether the gains in lamina association preferentially occur at these borders versus the interior of the domains. We observed that regions gaining lamina association following EZH2 inhibition, but not DNMT1 inhibition, reside within closer proximity to both LAD and compartment borders than would be expected by random chance (Supplementary Fig. 4h, i). Moreover, upon EZH2 inhibition, lamina association substantially increased at the borders of both LADs and compartments (Fig. 4h, Supplementary Fig. 4j). This trend was also evident when comparing changes in lamina association within H3K27me3 peaks at LAD borders versus within those not near LAD borders in the A compartment (iLADs) (Supplementary Fig. 4k).

To further evaluate PRC2-lamina antagonism, we assessed the effects of different PRC2 perturbations on lamina association. Instead of using the full LIMe-Hi-C workflow, we directly conducted lamina profiling by isolating and fragmenting genomic DNA from doxycycline-treated cells before performing bisulfite conversion followed by library preparation. This approach, which we term LIMe-ID, reveals LADs and CpG methylation with less sequencing depth required and could prove to be highly relevant to study their interdependence, especially given the observation that DNA hypomethylated domains in some cancers overlap with LADs (Supplementary Fig. 5a, b)^20,23^. We first performed LIMe-ID in a different K562 clonal line that expresses the M.CviPI-LaminB1 fusion after a 72 h treatment with vehicle, GSK343 (1 µM), or EED226 (5 µM), an orthogonal PRC2 inhibitor that targets EED and blocks allosteric activation of EZH2^56,57^. At a global level EED and EZH2 inhibitor treatments led to lamina GpC methylation signatures more similar to one another than to vehicle treatment (Fig. 4i). In line with our previous results, lamina association increased within PcG-B upon EED and EZH2 inhibition (Supplementary Fig. 5c-e). Consistent with these changes, an increase in lamina association both at LAD borders and within regions previously determined by LIMe-Hi-C to gain lamina contact upon EZH2 inhibition was observed (Supplementary Fig. 5f, g). As before, there were minimal changes in CpG methylation for both inhibitor treatments (Supplementary Fig. 5h).

To test the effects of acute EZH2 depletion on lamina positioning, we next generated a K562 clonal cell line with an inducible degradation tag (dTAG)^58^ fused to the endogenous N-terminus of EZH2 using CRISPR-Cas9 (Supplementary Fig. 6a). Addition of the dTAG-13 ligand led to the depletion of EZH2 and loss of H3K27me3 after 72 h (Supplementary Fig. 6a). LIMe-ID after acute depletion of EZH2 revealed gains in lamina association for Polycomb sub-compartments at levels similar to chemical inhibition of PRC2 (Fig. 4j, Supplementary Fig. 6b–d). Furthermore, these changes were enriched at LAD borders and correlated with H3K27me3 loss (Fig. 4k, Supplementary Fig. 6e, f) suggesting that H3K27me3 depletion is linked to lamina repositioning. Collectively, these data support the notion that H3K27me3 antagonizes lamina association, particularly at the boundaries of LADs and compartments that are enriched in this modification.

**Fig. 5:**
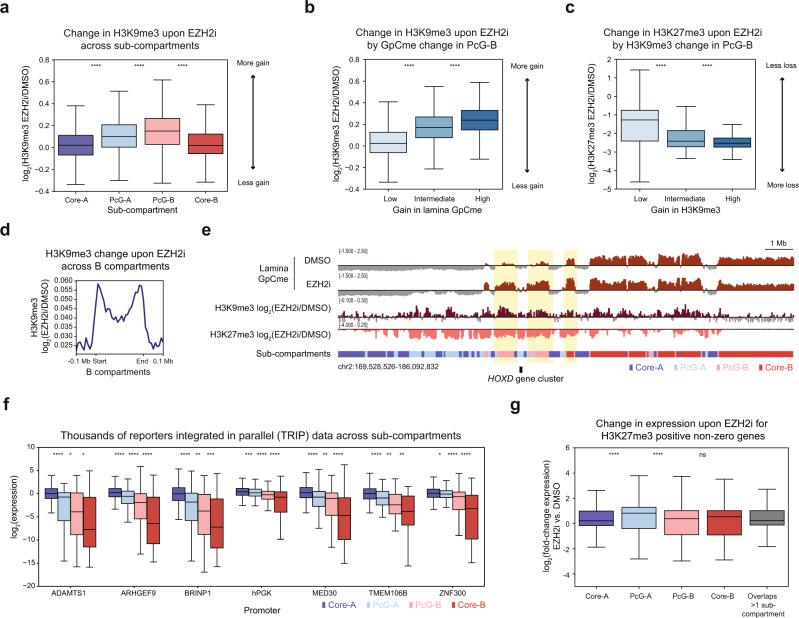
H3K27me3 antagonizes constitutive heterochromatin spreading. **a** Boxplot showing log_2_ fold-change H3K9me3 ChIP-seq signal between EZH2 inhibition and vehicle treatment for 50 kb bins (*y*-axis) across sub-compartments (*x*-axis). **b** Boxplot showing log_2_ fold-change in H3K9me3 levels between EZH2i and vehicle treatment for 50 kb bins (*y*-axis) segregated into three equally sized quantiles by change in *z*-score-normalized lamina GpC methylation (*x*-axis) for PcG-B. **c** Boxplot showing log_2_ fold-change in H3K27me3 levels between EZH2i and vehicle treatment for 50 kb bins (*y*-axis) segregated into three equally sized quantiles by log_2_ fold-change in H3K9me3 (*x*-axis) for PcG-B. **d** Aggregate profile plot of log_2_ fold-change H3K9me3 ChIP-seq signal between EZH2 inhibition and vehicle treatment (*y*-axis) across B compartment regions (*x*-axis). **e** Genome browser tracks of replicate-averaged *z*-score-normalized lamina GpC methylation levels for the LIMe-Hi-C data, log_2_ fold-change H3K9me3 ChIP-seq, and log_2_ fold-change H3K27me3 ChIP-seq signal between EZH2i and vehicle treatment near the *HOXD* locus. **f** Boxplot of log_2_(TRIP expression) (*y*-axis) across sub-compartments for promoters (*x*-axis). Outlier points are excluded. Published TRIP data is specified in Supplementary Table 1. **g** Boxplot of log_2_(fold-change expression) (*y*-axis) between EZH2 inhibition and vehicle treatment for genes that overlap with H3K27me3 peaks across sub-compartments (*x*-axis). In (**a**–**c**, **f**, **g**) the interquartile range (IQR) is depicted by the box with the median represented by the center line. Whiskers maximally extend to 1.5 × IQR (with outliers excluded). *P* values were calculated by a Mann-Whitney-Wilcoxon two-sided test and are annotated as follows: ns: not significant; *: 0.01 < *p* ≤ 0.05; **: 0.001 < *p* ≤ 0.01; ***: 0.0001 < *p* ≤ 0.001; ****: *p* ≤ 0.0001. Exact *p* values and the number of datapoints (*n*) compared are provided in the source data file.

We posited that H3K27me3-lamina antagonism may insulate subsets of facultative heterochromatin from the constitutive heterochromatin of the nuclear lamina environment. To assess this idea, we performed quantitative spike-in ChIP-seq for H3K9me3. Paralleling changes in lamina association, PcG-B gained the most H3K9me3 following EZH2 inhibition. In addition, increases in H3K9me3 were correlated with increases in lamina association within PcG-B (Fig. 5a, b). Furthermore, PcG-B regions that gained the most H3K9me3 lost the most H3K27me3 (Fig. 5c). Spreading of H3K9me3 into former H3K27me3 domains was also observed at the borders of B compartments and LADs as well as at Polycomb domains near the *HOXD* and *HOXA* loci (Fig. 5d, e, Supplementary Fig. 7a, b). Altogether, these results support a model where H3K27me3 at compartment borders prevents constitutive heterochromatin and lamina association from spreading into neighboring facultative heterochromatic regions^51^.

We next considered if heterochromatin segregation impacts transcription. To address this question, we analyzed published K562 data from thousands of reporters integrated in parallel (TRIP)^59^, an assay that assesses how chromatin context influences transcription by measuring the expression of the same reporter transcript along with its random genomic integration position. TRIP shows that PcG-B is on average a less repressive chromatin environment than Core-B, consistent with prior observations that the lamina is a strong mediator of gene silencing (Fig. 5f). Moreover, PcG-B is more repressive than PcG-A despite PcG-B regions possessing lower average levels of H3K27me3 (Fig. 2c). These patterns were mostly recapitulated when exclusively considering integrations in H3K27me3 ChIP-seq peaks within PcG-A and PcG-B (Supplementary Fig. 7c). These findings further support the notion that Polycomb sub-compartments and Core-B represent distinct forms of heterochromatin that have different capacities to silence gene expression^52^.

**Fig. 6:**
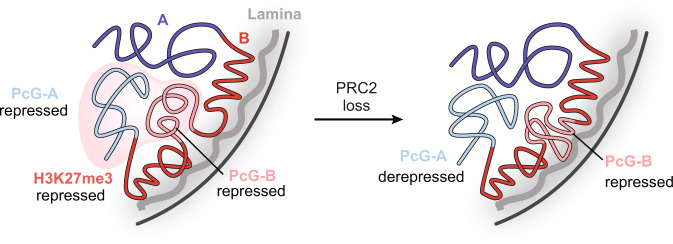
Proposed model of Polycomb-lamina antagonism. Schematic of epigenomic changes elicited by loss of PRC2.

Prompted by these observations, we considered if EZH2 inhibition led to sub-compartment-specific changes in gene expression. Upon EZH2 inhibition, we observed upregulation of 301 genes and downregulation of 151 genes (adjusted *p*-value < 0.05) (Supplementary Fig. 7d) (Supplementary Data 4). H3K27me3-marked genes within PcG-A showed the greatest increases in expression (Fig. 5g, Supplementary Fig. 7e). By contrast, H3K27me3-marked genes within PcG-B remained largely silenced despite both PcG-A and PcG-B genes being repressed prior to EZH2 inhibitor treatment (Fig. 5g, Supplementary Fig. 7e, f). These data indicate that EZH2 inhibition has compartment-specific effects on gene expression, with PcG-A genes poised for reactivation and PcG-B genes repressed by compensatory mechanisms—possibly by the nuclear lamina environment or H3K9me3 spreading (Fig. 6). Altogether, these results support the model that H3K27me3 insulates facultative heterochromatin from the nuclear lamina and the transcriptionally active portion of the A compartment. More broadly, these findings demonstrate how nuclear localization and compartmentalization modulate Polycomb-mediated gene repression.

## Discussion

In this work, we developed LIMe-Hi-C to simultaneously study lamina association, genome topology, and DNA methylation in a single experiment. The joint profiling of these features enables a global understanding of their interplay and functional consequences, which is often obscured through comparisons of independent datasets^21^. As we demonstrate, leveraging these multilayered global measurements with chemical inhibition of PRC2 allowed the identification of topologically distinct Polycomb sub-compartments across both the A and B compartments and the direct implication of H3K27me3 in their maintenance^31,32,34^. These findings are consistent with super-resolution imaging studies demonstrating that Polycomb-rich domains can self-associate and exclude other chromatin states^6^ and previous work partitioning LADs into facultative and constitutive heterochromatin domains^27,28^. Our work illustrates the multifaceted impact of H3K27me3 on compartmentalization, complementing previous findings on the distinct cohesin-independent nature of these interactions^13,14,36,37,60–62^.

Our studies uncover an antagonism between PRC2 and lamina association, especially for PcG-B regions lying at the borders of LADs and compartments, suggesting a major role of H3K27me3 in segregating heterochromatin subtypes near the nuclear periphery. These findings not only suggest how constitutive and facultative chromatin domains form topologically distinct sub-compartments but also highlight how their modes of lamina contact are independently regulated, with H3K27me3 opposing lamina contact and constitutive heterochromatin self-interaction. These observations were unexpected, as H3K27me3 has been hypothesized to mediate lamina association^10,24,53^. We propose that this discrepancy might arise from H3K27me3 facilitating peripheral positioning near the lamina, likely at LAD boundaries, but excluding direct contact. This model is consistent with the enrichment of H3K27me3 within the nuclear interior as well as the anti-correlation between lamina association and H3K27me3 levels observed in single-cell DamID experiments^17,63–65^. Furthermore, the antagonism between Polycomb-marked PcG-B and constitutively-marked Core-B regions supports prior findings detailing the differential regulation of facultative and constitutive heterochromatin evidenced upon lamin triple knock-out in mouse embryonic stem cells^27,28^.

The coordinated interplay among H3K27me3, the nuclear lamina, and compartmentalization has broad consequences on Polycomb-mediated repression of gene expression. While H3K27me3-marked genes within PcG-A are activated upon EZH2 inhibition, H3K27me3-marked genes within PcG-B remain largely silenced, consistent with PcG-B repositioning to the nuclear lamina and gaining H3K9me3 to a greater extent. These increases in constitutive heterochromatic features may prevent gene re-activation as both the lamina and HP1α, which binds H3K9me3 and interacts with nuclear lamina proteins, are implicated in gene repression^1,59,66^. Although Polycomb and the nuclear lamina both mediate gene silencing within the B compartment, maintaining two separate forms of heterochromatin may prevent constitutive silencing of Polycomb-genes, enabling their expression in certain contexts (e.g., differentiation)^67,68^. Moreover, the less transcriptionally permissive nature of PcG-B compared to PcG-A reflects how spatial segregation within A compartment regions might poise Polycomb-repressed genes for reactivation. These findings have implications on the functional impact of PRC2 inhibitors, many of which are under clinical evaluation. Altogether, our study illustrates how the complex interplay among Polycomb, A/B compartmentalization, and the nuclear lamina regulates genome structure and gene expression, more broadly highlighting how the cell integrates many regulatory inputs to ensure that gene expression programs are not only stable but also tunable.

## Methods

### Cell culture and lentivirus production

K562 was obtained from ATCC. HEK 293 T was obtained from Life Technologies. K562 was authenticated by Short Tandem Repeat profiling (Genetica) and routinely tested for mycoplasma (Sigma-Aldrich, MP0035)). All cell lines were cultured in a humidified 5% CO_2_ incubator at 37 °C with media supplemented with 100 U/mL penicillin and 100 µg/mL streptomycin (Life Technologies, 15070063). K562 was cultured in RPMI-1640 (Life Technologies, 11875119) with 10% fetal bovine serum (FBS) (Peak Serum, PS-FB2). HEK 293 T was cultured in DMEM (Life Technologies, 1195073) with 10% FBS (Peak Serum, PS-FB2). For lentivirus production, expression plasmids were co-transfected with *GAG/POL* and *VSVG* plasmids into HEK 293 T cells using FuGENE HD (Promega, E2312). Media was exchanged after 6–10 h, and the viral supernatant was collected 48–72 h after transfection and filtered (0.45 µm). K562 was transduced by spinfection at 2000 g for 90 min at 37 °C with 8 µg/mL polybrene (Santa Cruz Biotechnology, SC-134220). To generate the M.CviPI overexpression bulk cell lines, 48 h post-transduction, geneticin (Life Technologies) selection was carried out for approximately one week at 0.6 mg/mL. Single-cell clones were isolated from the bulk cell line through fluorescence-activated cell sorting, and the expression of the fusion was validated through immunoblot.

### Mammalian overexpression constructs

The Lamin B1 open reading frame (ORF) was subcloned from mCherry-LaminB1-10, a gift from M. Davidson (Addgene #55609), and the M.CviPI ORF with a C-terminal glycine-serine (GS) linker (GGSGGGS) was ordered as a gene block (Integrated DNA Technologies). An N-terminal FLAG tag and a nuclear localization signal (NLS) along with the M.CviPI ORF were then cloned N-terminal to Lamin B1 into pENTR (Invitrogen) using Gibson cloning (New England Biolabs, E2621X). The final overexpression construct for FLAG-NLS-M.CviPI-LaminB1 in pInducer20 (Invitrogen) was obtained through a recombination reaction using Gateway cloning (LR Clonase™ II, Thermo Fisher, 11-791-020) between the M.CviPI-pENTR construct and pInducer20. For the LIMe-ID experiments with EED and EZH2 inhibition, the M.CviPI-LaminB1 plasmid without the FLAG-NLS sequence was employed instead and cloned in an analogous manner.

### Generating dTAG-EZH2 knock-in

EZH2 homology arms of 1 kb were amplified from K562 genomic DNA and cloned into the ph1 backbone along with Puro-P2A-dTAG, sub-cloned from pCRIS-PITChv2-Puro-dTAG, a gift from J. Bradner (Addgene # 91793). The sgRNA, 5’-GAGAAGGGACCAGTTTGTTGG-3’ that was previously validated for N-terminal EZH2 knock-in^69^ was cloned into PX459 by restriction enzyme digestion. Wild-type K562 cells were transfected with both the donor and guide plasmids by nucleofection (Lonza, program T016). Cells were selected 3 days after transfection with puromycin (2 µg/mL) (Thermo Fisher Scientific, A1113803) for a duration of 5 days. Single-cell clones were isolated from the bulk cell line through fluorescence-activated cell sorting and validated by western blot and Sanger sequencing. The Flag-NLS-M.CviPI-LaminB1 construct was then transduced into this clonal knock-in cell line, and selection was carried out with geneticin as specified above. The cell line resulting from this transduction was then used directly for subsequent LIMe-ID experiments.

### Immunoblotting

Fusion protein overexpression was induced by treatment with doxycycline (dox) (Sigma Aldrich) at a concentration of 1 µg/mL for the specified amount of time. Pellets were harvested for western blot analysis and washed with PBS (Corning, 21-040-CV). Cells were subsequently lysed on ice using RIPA buffer (Boston BioProducts, BP-115-250) supplemented with fresh HALT Protease Inhibitor with EDTA (Thermo Fisher Scientific, 78429), and the lysates were clarified through centrifugation. For histone immunoblotting, histones were extracted according to the recommended manufacturer’s one-step protocol using the Histone Extraction Kit (Active Motif, 40028). The protein concentration of the lysates was determined using the BCA Protein Assay Kit (Thermo Fisher Scientific, 23225). Immunoblotting was performed according to standard procedures. To assess histone loading, the blot was stripped with Restore Western Blot Stripping Buffer (Thermo Fisher Scientific, 20159) for 30 min prior to re-probing for histone H3. The primary antibodies used for immunoblotting are as follows: GAPDH (1:5000, Santa Cruz Biotechnology sc-477724); monoclonal anti-FLAG M2 (1:1000 and 1:5000, Sigma-Aldrich F1804); Tri-Methyl-Histone H3 Lys27 (1:10000, Cell Signaling Technology C36B11); Histone H3 (1:10000, Cell Signaling Technology 9715 S); and EZH2 (1:4000, Cell Signaling Technology 5246).

### Cell growth assays

Flag-NLS-M.CviPI-Lamin B1 K562 cells were plated in triplicate at a density of 4000/well in a 96-well plate. Drug or vehicle (DMSO, Sigma Aldrich, D8418) were dosed at the specified concentration for 3 days. After 3 days of treatment, cell viability was measured using CellTiter-Glo (Promega, G7572) with the luminescence detector on the SpectraMax i3x (Molecular Devices) plate reader with the SoftMax Pro (version 6.5.1) software, and data were processed using Prism.

### Inhibitor treatment for Hi-C and RNA-seq

The Flag-NLS-M.CviPI-LaminB1 clonal cell line was seeded at a density of 125 K/mL and treated with 10 µM GSK3482364 (synthesized as previously described)^48,49^, 1 µM GSK343 (Selleck Chemicals, S7164), or vehicle (0.1% DMSO (Sigma Aldrich, D8418)) for a total of three days. Two days after the start of the inhibitor or vehicle treatment, doxycycline (Sigma Aldrich) was added to the cells at a final concentration of 1 µg/mL for the remaining 24 hours of the drug treatments. For the no induction condition, blank media was added in place of doxycycline. Upon completion of the drug treatments, pellets were taken for Hi-C, LIMe-Hi-C, and RNA-seq in triplicate.

### Hi-C and LIMe-Hi-C

Cells were treated in triplicate with either GSK343, GSK3482364, or vehicle and induced with doxycycline as specified above. In situ Hi-C was performed according to a published protocol^11^. Briefly, 4 ⨯ 10^6^ cells were fixed at a density of 10^6^ cells/mL in media containing 10% FBS and 1% formaldehyde (Sigma Aldrich, F8775) for 10 min with rotation. Formaldehyde was quenched with glycine (final concentration, 0.2 M), and cell pellets were washed with PBS and flash frozen. Nuclei were lysed, and overnight digestion was performed using 200 U of DpnII (New England Biolabs, R0543L) at 37 °C with shaking in NEB buffer 3.1 (New England Biolabs, B7203). Heat inactivation was performed by incubating the samples at 65 °C for 20 min. Digested ends were filled in with biotinylated dATP (Life Technologies, 19524-016) for 30 min at 37 °C, and in situ ligation was performed with T4 DNA ligase (New England Biolabs, M0202L) for 6 hours at room temperature with rotation. Samples were digested with Proteinase K (Invitrogen, 25530015) at 55 °C for 30 min and heated overnight at 68 °C to reverse the crosslinks. DNA was purified through an ethanol precipitation and sheared to a size of 300-500 bp with sonication using the Covaris sonicator (S220). DNA was size-selected following shearing using a 0.55x followed by a 0.70x double-SPRI magnetic bead purification (Omega Bio-Tek, M1378-01). At this point, the DNA was split in half for bisulfite (LIMe) and traditional in situ Hi-C, if performed. For the traditional in situ Hi-C workflow, biotinylated fragments were isolated using T1 streptavidin beads (Life Technologies, 65601), and library preparation was performed on beads according to published precedent^11^. Specifically, beads were bound in Binding buffer (10 mM Tris-HCl pH 7.5, 1 mM EDTA, 2 M NaCl) for 15 min at room temperature. Beads were washed twice with Tween washing buffer (5 mM Tris-HCl pH 7.5, 0.5 mM EDTA, 1 M NaCl, 0.5% Tween-20) and once with 1X NEB T4 DNA ligase buffer (New England Biolabs, B0202), and end repair was performed at room temperature for 30 min using NEB T4 PNK (New England Buffer, M0201), NEB T4 DNA polymerase I (New England Biolabs, M0203), and NEB DNA polymerase I Large (Klenow) Fragment (New England Biolabs, M0210). Beads were subsequently washed twice with Tween washing buffer and once with NEB Buffer 2 (New England Biolabs, B7002). A tailing was performed using NEB Klenow Fragment (3’−5’ exo-) (New England Biolabs, M0212). Beads were subsequently washed twice with Tween washing buffer and once with NEB Quick Ligation Reaction Buffer (New England Biolabs, B6058). Adapter ligation was performed using NEB DNA Quick Ligase (New England Biolabs, M2200) with KAPA single-indexed barcoded adapters (Kapa Biosystems, KR1317). Beads were subsequently washed twice with Tween washing buffer and once with 10 mM Tris-HCl. PCR was then performed on beads using the NEB Phusion polymerase (New England Biolabs, M0530). The supernatant was separated from the beads, and DNA was purified using a 0.7 X SPRI purification (Omega Bio-Tek, M1378-01) to obtain final sequencing libraries. The preparation of bisulfite (LIMe) Hi-C libraries was performed according to the Hi-Culfite protocol^40^. Specifically, 0.5-1 µg of DNA was bisulfite-converted with the EpiTect Fast Bisulfite Conversion Kit (Qiagen, 59824) using two columns per sample according to the manufacturer’s protocol with the following modifications: the two 60 °C bisulfite conversion incubations were extended from 10 min to 20 min each, and DNA was purified without carrier RNA. Following bisulfite conversion, the samples were eluted in a final volume of 20 µL, and 15 µL of material was used. Biotinylated fragments were purified using C1 streptavidin beads (Life Technologies, 65001). Specifically, 15 µL of streptavidin C1 beads were added to each sample, and the biotinylated DNA was allowed to incubate for 10 min with the beads at 55 °C in 1x denaturing buffer (10 mM Tris pH 7.5, 5 mM EDTA, 500 mM LiCl, 0.5% Igepal CA630, 0.2% SDS, 4 M urea). Following the incubation, the streptavidin beads were washed twice with prewarmed denaturing buffer at 55 °C for 2 min and once with 10 mM Tris-HCl pH 7.5. DNA was eluted in 15 µL of Tris pH 7.5 for 5 min at 95 °C and subsequently quantified using ssDNA Qubit quantification. LIMe-Hi-C libraries were then generated from 25-50 ng of input DNA using Accel-NGS-methyl-Seq Kit (Swift Biosciences, 30024) according to the manufacturers protocol with 9 cycles of amplification using the Swift Single Indexing Primers Set A (Swift Biosciences, X6024). Care was taken to avoid freeze-thaw cycles once the DNA was bisulfite-converted prior to library preparation. Quality of the libraries were assessed using a MiSeq Genome Analyzer (Illumina) with 75 bp paired-end reads. Libraries were subsequently sequenced on a NovaSeq S4 kit (Illumina) with 150 bp paired-end reads to a sequencing depth of around 600 million reads per sample.

### Quantitative ChIP-seq

ChIP was performed in duplicate for Flag-NLS-M.CviPI-Lamin B1 cells treated with DMSO or GSK343 and induced with doxycycline as described above^70^. Specifically, cells were fixed at a density of 10^6^ cell/mL in media containing 10% FBS (Peak Serum, PS-FB2) and 1% formaldehyde (Sigma Aldrich, F8775) for 10 min with rotation and quenched with 0.2 M glycine. 10 million cells per condition were lysed on ice for 10 min in 2 mL of ChIP lysis buffer (50 mM Tris-HCl pH 7.5, 0.1% SDS, 150 mM NaCl, 0.25% Triton X-100, 5 mM EDTA) with HALT protease inhibitors (Thermo Fisher Scientific, 78429). Cells were sonicated with the Branson Sonicator, and the supernatant was clarified by centrifugation. Spike-in Drosophila S2 chromatin (Active Motif, 08221011) at a concentration of 10 ng/million cells and spike-in antibody (Active Motif, 61686) at a concentration of 0.4 µg/million cells were added following lysis and sonication but prior to immunoprecipitation. The sample was then split into different immunoprecipitation conditions. H3K27me3 immunoprecipitation was performed on 4 million cells with 10 µL of H3K27me3 antibody (Cell Signaling, C36B11 Lot #19). H3K9me3 immunoprecipitation was performed on 3 million cells with 8 µL of H3K9me3 antibody (Cell Signaling, 13969 S Lot #3). Immunoprecipitations were carried out overnight at 4 °C with rotation. Protein G Dynabeads (Thermo Fisher Scientific, 10004D) were added to samples and incubated at 4 °C with rotation for 3 hours. Beads were then washed twice with ice-cold RIPA wash buffer (10 mM Tris-HCl pH 8.1, 0.1% SDS, 150 mM NaCl, 0.1 NaDOC, 1% Triton X-100, 1 mM EDTA), washed once with ice-cold LiCl wash buffer (10 mM Tris-HCl, pH 8.1, 250 mM LiCl, 0.5% Triton X-100, 0.5% NaDOC) and eluted in 100 µL of Elution buffer (10 mM Tris-HCl pH 8.0, 0.1% SDS, 150 mM NaCl, 1 mM EDTA) at 65 °C for 1 hour. Samples were then treated with 50 µg of RNAse (Sigma Aldrich, 10109142001) for 30 min at 37 °C. Next, 25 µg of Proteinase K (Invitrogen, 25530015) was added, and the samples were allowed to incubate overnight at 63 °C with agitation. DNA was subsequently purified by a 2X SPRI (Omega Bio-Tek, M1378-01). Sequencing libraries were then prepared from 2.5 ng of DNA by performing end-repair with the End-it DNA End-Repair Kit (Lucigen, ER81050), followed by A tailing with NEB Klenow Fragment (3’−5’ exo-) (New England Biolabs, M0212), adapter ligation with NEB DNA Quick Ligase (New England Biolabs, M2200), and PCR using the 10X KAPA HiFi HotStart Ready Mix (Kapa Biosystems, KK2611). Samples were sequenced using a NovaSeq SP kit (Illumina) with 50 bp paired-end reads at a sequencing depth of approximately 20 million reads per sample.

### RNA-seq

In triplicate separate cultures, cells were treated with GSK343 or vehicle and induced with doxycycline as described above. Total RNA was isolated using the RNeasy Plus Mini Kit (Qiagen, 74104). Library preparation was performed using the Quantseq 3’ mRNA-Seq Library Prep Kit FWD (Lexogen, 015.24) according to the manufacturer’s protocol. The samples were sequenced with a NovaSeq SP kit (Illumina) with 50 bp single-end reads at a sequencing depth of approximately 10 million reads per sample.

### LIMe-ID

For inhibitor LIMe-ID experiments, M.CviPI-Lamin-B1 cells were treated in duplicate with 1 µM GSK343 (Selleck Chemicals, S7164), 5 µM EED226 (Selleck Chemicals, S8496), or vehicle (0.1% DMSO (Sigma Aldrich, D8418)) for a total of 3 days and induced with doxycycline as specified above. For degradation LIMe-ID experiments, Flag-NLS-M.CviPI-Lamin-B1 dTAG-EZH2 knock-in cells were treated in duplicate with either 500 nM dTAG-13 (Sigma Aldrich, SML2601) or vehicle (0.1% DMSO (Sigma Aldrich, D8418)) for a total of 3 days and induced with doxycycline as specified above. Cell pellets of 1 million cells each were harvested by centrifugation and washed with PBS (Corning, 21-040-CV). DNA was purified through QIAamp DNA blood mini kit (Qiagen, 51106) with the addition of RNAse (Qiagen, 19101) and sheared to a size of 400 bp in 50 µL by sonication using the Covaris sonicator (S220). DNA was subsequently bisulfite converted with the EpiTect Fast Bisulfite Conversion Kit (Qiagen, 59824) according to the manufacturer’s protocol with the following modifications: the two 60 °C bisulfite conversion incubations were extended from 10 min to 20 min each, and DNA was purified without carrier RNA. DNA was quantified, and libraries were then generated from 25-50 ng of input DNA using Accel-NGS-methyl-Seq Kit (Swift Biosciences, 30024) according to the manufacturers protocol with 9 cycles of amplification using the Swift Single Indexing Primers Set A (Swift Biosciences, X6024). Samples were sequenced using a NovaSeq SP kit (Illumina) with 50 bp paired-end reads at a sequencing depth of approximately 30 million reads per sample.

### Hi-C and LIMe-Hi-C data processing

In situ Hi-C data were processed according to the SLURM version of Juicer pipeline (version 1.6) with JuicerTools (version 1.22.01)^71^. The LIMe-Hi-C data were processed utilizing the CPU version of the JuiceMe (version 1.0.0) pipeline developed for analyzing bisulfite Hi-C data using JuicerTools (version 1.22.01) with the following modifications^40,71^. Reads were aligned to the hg38 reference genome containing no ALT contigs through bwa-meth (version 0.2.2)^72^. In addition, in order to identify both GpC methylation and CpG methylation, the Biscuit pileup program (version 0.3.16)^73^ was utilized with the parameters “-q 12, -N”. To differentiate exogenous and endogenous DNA methylation, we take advantage of the fact that these modifications occur within different base contexts. Specifically, endogenous DNA methylation occurs in CpG contexts while M.CviPI introduces DNA methylation in GpC contexts. To differentiate the two, we utilize the Biscuit analysis pipeline and specify that only “GCH” are considered for lamina signal detection, and only HCG sites are considered for endogenous DNA methylation detection (H = ATC). By only considering GCH and HCG, we exclude GpCpG sites where the methylation signal would be ambiguous. In particular, to obtain position-specific information on GpC and CpG methylation, the Biscuit vcf2bed program was employed with the parameters “-k 3” and “-t gch” or “-t hcg” to exclude GpCpG sites. Juicebox (version 1.11.08)^71^ was used for exploratory data analysis and for plotting Hi-C contact maps. The bedgraph files generated from the methylation analysis were converted to bigwig format utilizing the UCSC bedGraphToBigWig function (version 4), which was subsequently imported into Integrated Genome Browser (version 2.5.3)^74^ and Juicebox (version 1.11.08) for visualization or used as input for further analysis. Subsequent data processing was performed in Python (version 3.7.0) (www.python.org) and R (version 3.6.1) (www.r-project.org) with Scipy (version 1.2.1), seaborn (version 0.11.1), and matplotlib (version 2.0.3).

### LIMe-Hi-C per-read analysis

Data were processed according to the JuiceMe pipeline and the per-read analysis scripts (https://github.com/aidenlab/JuiceMe/tree/master/Analysis) with modifications detailed below. The Biscuit epiread program with the parameters “-N -m 0 -l 0 -c -u -p” was utilized to create a file detailing both the CpG and GpC methylation status of individual reads from the methylation.bam output of the JuiceMe pipeline (Biscuit, version 0.3.16)^73^. A custom awk script was employed to format the methylation status of the reads appropriately. For the contact matrix file, only intrachromosomal contacts from different fragments with a MAPQ score >0 were considered. After filtering the contact matrix for reads from a chromosome of interest, they were reassigned to a chromosomal value by their methylation status. For example, a read originally mapping to chromosome 1 would be assigned to “chr1m” if it were methylated and “chr1u” if it were unmethylated. A read was considered CpG methylated if 50% of CpG sites within the read were methylated. A read was considered GpC methylated if 10% of GpC sites within the read were methylated. Contacts were then sorted according to their methylation status. To reduce bias from fragments arising from directly neighboring regions, contacts were discarded if its reads mapped to within less than 1 kb of one another. Finally, these contacts were outputted to a hic matrix file using the Juicer tools “pre” function. Matrix files detailing contacts by methylation status were then generated using the Juicer tools “dump” function. The M matrix file contains counts for bins where both read pairs are methylated while the U matrix file contains counts for bins where both read pairs are unmethylated. The Y matrix file is the unsymmetric matrix containing counts for contacts where the read at index i is methylated but the read at index j is not. These files were then loaded into python and analyzed as previously described (see Supplementary Zip File)^40^.

The M matrix for CpG and GpC methylation was directly visualized in Juicebox (version 1.11.08) and displayed in Fig. 1. Observed minus expected co-methylation is plotted in heatmap form and is defined as previously described:

$${{{{{{\rm{OE}}}}}}}_{{{{{{\rm{ij}}}}}}}=(({{{{{{\rm{M}}}}}}}_{{{{{{\rm{ij}}}}}}}+{{{{{{\rm{U}}}}}}}_{{{{{{\rm{ij}}}}}}})/{{{{{{\rm{T}}}}}}}_{{{{{{\rm{ij}}}}}}}){-}[\left({{{{{{\rm{A}}}}}}}_{{{{{{\rm{i}}}}}}}\ast {{{{{{\rm{A}}}}}}}_{{{{{{\rm{j}}}}}}}\right)+(1-{{{{{{\rm{A}}}}}}}_{{{{{{\rm{i}}}}}}})(1-{{{{{{\rm{A}}}}}}}_{{{{{{\rm{j}}}}}}})]$$

The total number of contacts, T_ij_, is defined as:

$${{{{{{\rm{T}}}}}}}_{{{{{{\rm{ij}}}}}}}={{{{{{\rm{M}}}}}}}_{{{{{{\rm{ij}}}}}}}+{{{{{{\rm{Y}}}}}}}_{{{{{{\rm{ij}}}}}}}+{{{{{{\rm{Y}}}}}}}_{{{{{{\rm{ji}}}}}}}+{{{{{{\rm{U}}}}}}}_{{{{{{\rm{ij}}}}}}}$$

The average methylation of a given locus, A_i_, is defined as:

$${{{{{{\rm{A}}}}}}}_{{{{{{\rm{i}}}}}}}={\sum}_{{{{{{\rm{j}}}}}}}(({{{{{{\rm{M}}}}}}}_{{{{{{\rm{ij}}}}}}}+{{{{{{\rm{Y}}}}}}}_{{{{{{\rm{ij}}}}}}})/{{{{{{\rm{T}}}}}}}_{{{{{{\rm{ij}}}}}}}).$$

To obtain information on positional dependence of observed methylation across sub-compartments, the observed methylation matrix is defined as:

$${{{{{{\rm{O}}}}}}}_{{{{{{\rm{ij}}}}}}}=(({{{{{{\rm{M}}}}}}}_{{{{{{\rm{ij}}}}}}}+{{{{{{\rm{Y}}}}}}}_{{{{{{\rm{ij}}}}}}})/{{{{{{\rm{T}}}}}}}_{{{{{{\rm{ij}}}}}}})$$

and was min-max normalized by using the average methylation in the A compartment as the min value and the average methylation in the B compartment as the max to correct for differences in dynamic range between samples and vice versa for CpG methylation. The average value of this matrix across 50 kb bins for interactions between compartments or sub-compartments is displayed in heatmap form. Summary boxplots across all chromosomes of these values for PcG-B and Core-B are included. For locus-specific lamina association analysis, the average observed lamina GpC methylation for all intrachromosomal interacting bins for a given locus was calculated for each sub-compartment or compartment of interest and the specified log_2_ ratio was plotted.

### LIMe-ID Analysis

LIMe-ID analysis was performed analogously to the LIMe-Hi-C analysis except that reads were aligned utilizing the Biscuit alignment pipeline with the following parameters: “biscuit align -t 16 -R ‘@RG\tID’ Read1.fastq.gz Read2fastq.gz | samblaster | samtools sort -o sample.bam -O BAM –” (Biscuit, version 0.3.16)^73^. In addition, to obtain position-specific information regarding GpC and CpG methylation, the Biscuit vcf2bed program (version 0.3.16) was employed with the parameters “-k 1” instead of “-k 3” to account for the lower sequencing depth utilized for LIMe-ID.

### Sub-compartment identification and integration of published data

GpC methylation, CpG methylation, and principal component 1 values were averaged across 50 kb bins utilizing the multiBigwigSummary function (deepTools, version 3.4.3). For CpG methylation analysis, CpG islands were excluded by subtracting CpG sites that overlapped with previously annotated islands from UCSC [http://hgdownload.cse.ucsc.edu/goldenpath/hg38/database/cpgislandExt.txt.gz], which were padded by 2 kb using Bedtools2 (version 2.26.0)^75^. These values were *z*-score normalized, and the average values across the three replicates for the DMSO-induced (vehicle) condition were calculated for every 50 kb bin. Regions with an average |*z-*score | > 3 were excluded from the classification. *K*-means clustering and visualization were performed in R (version 3.6.1) with pheatmap (kmeans = 4) (version 1.0.12)^76^ across the average *z*-score-normalized GpC methylation, CpG methylation, and principal component 1 values to classify each 50 kb genomic bin into a sub-compartment. Published ChIP-seq and DamID data (see Supplementary Table 1) as well as quantitative ChIP-seq data, processed as described below, were similarly averaged across 50 kb bins using the multiBigwigSummary function to determine sub-compartment enrichment and correlation with LIMe-Hi-C features.

### Quantitative ChIP-seq analysis

Reads were aligned to a composite reference genome for dm6 and hg38 utilizing bowtie2 (version 2.3.2)^77^ according to the Spiker analysis framework (version 1.03)^78^. Aligned reads were sorted using samtools (version 0.1.19)^79^, and the Spiker split_bam.py script was run to calculate scaling factors and to split reads into an alignment file containing reads specifically aligning to the hg38 genome (Spiker, version 1.0.3)^78^. This alignment file was then converted into a spike-in normalized bigwig file for further analysis and visualization utilizing the deepTools bamCoverage command with the following parameters: -binSize 50 --normalizeUsing RPKM --ignoreDuplicates --ignoreForNormalization chrM --extendReads --scaleFactor. Peaks for individual H3K27me3 replicates were called utilizing MACS2 “callpeak” function (version 2.1.1.20160309) with the following parameters: -f BEDPE -B -g hs—broad—broad-cutoff 0.2. Regions within 10 kb of one another were merged utilizing the Bedtools2 merge function -d 10000 (Bedtools2, version 2.26.0). Finally, individual replicate peaks were subsequently combined utilizing Bedtools2 merge. For bin level analysis, the H3K27me3 signal value was averaged across 50 kb bins utilizing the multiBigwigSummary function (deepTools, version 3.4.3)^80^. To generate metaprofile plots comparing treatment conditions, the “log2” option in the bigwigCompare function was used prior to plotting with the plotHeatmap function (deepTools, version 3.4.3)^80^.

### Identifying LIMe LADs and differential lamina GpC methylated regions

Genome-wide 50 kb GpC methylation data was calculated as specified above and *z*-score transformed. For every replicate, the Bumphunter package (version 1.34.0)^45,46^ in R was subsequently used to identify GpC methylated (*i.e*., lamina attached) regions with a threshold of 0. Regions that contained 2 or less contiguous bins (<100 kb) were removed. The set of high confidence LIMe LADs were then defined to be those regions present in at least two of the three replicates. The overlap between LIMe LADs and DamID LADs was calculated for all regions with a sub-compartment designation using pybedtools (version 0.8.1)^81^ “intersect” and “subtract” functions.

To identify differential lamina GpC methylated regions upon either EZH2 or DNMT1 inhibition, a *t*-statistic was calculated by comparing the *z*-score-normalized GpC methylation data of three replicates for the inhibitor samples to that of the vehicle-treated samples. A threshold of 4 was utilized to identify differential regions upon EZH2i and threshold of 2 was utilized to determine differential regions upon DNMT1i. Differential regions that contained 2 or less contiguous bins (<100 kb) were excluded from the set of differential regions.

### Principal component analysis and contact frequency analysis

The JuiceMe merged_nodups.txt output file containing a list of paired contacts was reformatted and converted to the HiCSummary format (Homer, version 4.10)^82^. Homer tag directories were generated, and the runHiCpca function with the parameters “-res 50000 -superRes 50000 -std 4 -min 0.25” was used to calculate the first principal component of the Hi-C contact matrix.

To perform the A/B ratio and observed/expected contact frequency analysis, Knight-Ruiz normalized 50 kb binned coordinate sorted matrices were generated using the JuicerTools “dump” function. A/B ratio was calculated as previously described^34^ and represents the log of the ratio of the average observed/expected intra-chromosomal interaction frequency of a given 50 kb region with A compartment bins relative to B compartment bins. Specifically, the A/B ratio is defined as the log_2_(average observed/expected interaction frequency with intrachromosomal A regions) - log_2_(average observed/expected interaction frequency with intrachromosomal B regions). As input compartment designations, the average of principal component 1 across the three replicates was calculated. The bin was assigned to the A compartment if this value was > 0 and assigned to the B compartment if this value was < 0^34^. A similar analysis was performed for the sub-compartment designations except sub-compartment labels were utilized as input regions to calculate average observed/expected contact frequencies.

### H3K27me3-ordered sub-compartment interaction plots

Contact files (.hic) were converted to the cool format using the hic2cool function (version 0.8.3)^83^. Expected contact frequencies were calculated through the following command: “cooltools compute-expected—weight-name KR -t cis” (cooltools, version 0.4.0)^84^. For H3K27me3-ordered sub-compartment interaction plots, the genome was divided into 100 quantiles with the number of quantiles within a sub-compartment reflective of the relative size of the sub-compartment. Specifically, 50 kb regions were assigned to a quantile based on H3K27me3 levels within their respective sub-compartment, and these designations were used as input for the following command: “cooltools compute-saddle -t cis—weight-name KR—quantiles -n 100” with chromosomal arms as input regions. These values were log_2_-transformed and averaged over replicates. Standard plotting functions (Python version 3.7.0) were then employed to display the calculated saddle output. To compare inhibitor to vehicle treatment, the difference of the averaged log_2_-transformed saddle data across replicates was calculated and displayed.

### Measuring the distance of regions to borders and their overlap with sub-compartments and LAD sub-types

Confident B compartment calls were determined through findHiCCompartments (Homer, version 4.10) for all three DMSO-induced replicates with the parameter “-opp”. Regions present in at least two of the three replicates were used to define the set of high confidence B regions. Borders were specified to be the single base pair coordinate at the boundaries of the B compartment domains. Boundary coordinates were specified in a similar manner for the LIMe LADs. The distance of every differential region to a border was determined through the pybedtools (version 0.8.1)^81^ “closest” function, and the median across the whole set of regions was calculated. To determine how this median distance compared to that which would be expected by random chance, the coordinates of the differential regions were shuffled within a chromosome with the pybedtools (version 0.8.1) “shuffle” function, and the same calculation was performed. This random shuffling was repeated 1000 times. An analogous calculation was performed to determine overlap of differential regions with the sub-compartments except that the pybedtools (version 0.8.1) Jaccard metric was utilized in place of the “closest” function. The ratio of the observed Jaccard metric to the simulation average Jaccard metric was calculated to measure the enrichment of a set of regions within a specific sub-compartment. To calculate base-pair overlap with H3K27me3 peaks, LADs, and LAD sub-types, the pybedtools (version 0.8.1) “intersect” function was employed. For the LAD sub-type analysis, published coordinates as defined in KBM7 (https://osf.io/dk8pm/wiki/home/) were used, and these regions were converted to hg38 coordinates utilizing the NCBI genome remap tool.

### Metaprofile plots

Aggregate heatmaps were generated utilizing the computeMatrix and plotHeatmap functions (deepTools, version 3.4.3) for specified genomic regions with bigwig files for H3K27me3 and H3K9me3 ChIP-seq enrichment and differential GpC methylation. Differential GpC methylation was calculated by subtracting *z*-score-normalized GpC methylation signal averaged across the replicates between vehicle and inhibitor treatments.

The genomic distribution of the sub-compartments was determined by utilizing the computeMatrix and plotProfile functions (deepTools version 3.4.3) with high confidence B compartment and A compartment regions (calculated as specified above for B compartments except using findHiCCompartments without the “-opp” parameter). The presence or absence of a sub-compartment bin at a given location was specified, and regions with no coverage for a sub-compartment were given a zero score for enrichment using the “missingDataAsZero” parameter in the computeMatrix function.

### RNA-seq processing and differential gene expression analysis

RNA-seq data were processed according to the Quantseq 3’ mRNA-Seq Library prep recommended analysis pipeline through alignment to the Ensemble hg38 transcriptome. HTSeq (version 0.11.2)^85^ was used to generate count files. DESeq2 (version 1.28.1)^86^ with R (version 4.0.2) was employed for the differential expression and FPM analysis, and BioMart (version 2.44.1)^87^ with R was used to map genomic ranges of the transcripts. Bedtools2 (version 2.26.0) map was used to annotate genes with sub-compartments, and Bedtools2 coverage was used to determine H3K27me3 status, where a gene was considered H3K27me3-positive if at least 1 base pair overlapped with an H3K27me3 peak. Genes that did not overlap any of the sub-compartments (<1% of non-zero genes) were excluded from the analysis.

### TRIP expression analysis

Published K562 TRIP data utilized in this study are specified in Supplementary Table 1. Overlap of the promoter integrations with sub-compartments and H3K27me3 peaks was determined through Bedtools2 (version 2.26.0) intersect. The log_2_ transformation of the reported expression value is depicted in the boxplots.

### Statistical methods

Statistical tests and parameters used are reported directly in the figure legends. The definition of center, dispersion, and precision measurements (mean + /− s.d. or s.e.m.) are reported in the figures and figure legends. The Spearman correlation was calculated with the stats.spearmanr function (SciPy, version 1.2.1). The *t*-statistic metric was determined with the two-sided stats.ttest_ind function or the one-sided ttest_1samp function (SciPy, version 1.2.1) assuming unequal variance. For all boxplots, the median and the interquartile range (IQR) are depicted by the box, and the whiskers maximally extend to 1.5 × IQR. Exclusion of outlier points outside this range is specified in the figure legend. Linear regression was performed using the stats.linregress function (SciPy, version 1.2.1) with the R^2^ value specified in the figure. For all plots, measurements averaged across replicates are employed unless otherwise stated.

### Reporting Summary

Further information on research design is available in the Nature Research Reporting Summary linked to this article.

## Supplementary information

Supplementary Information

Description of additional Supplementary File

Supplementary Data 1

Supplementary Data 2

Supplementary Data 3

Supplementary Data 4

Supplementary Software

Reporting Summary

## Data Availability

The data that support this study are available from the corresponding authors upon reasonable request. All sequencing data has been deposited on NCBI Gene Expression Omnibus under the super series accession code GSE180230. The LIMe-Hi-C data generated in this study have been deposited in the NCBI Gene Expression Omnibus under accession code GSE180228. The RNA-seq data generated in this study have been deposited in the NCBI Gene Expression Omnibus under accession code GSE180229. The ChIP-seq data generated in this study have been deposited in the NCBI Gene Expression Omnibus under accession code GSE198613. The LIMe-ID data generated in this study have been deposited in the NCBI Gene Expression Omnibus under accession code GSE198615. The K562 H3K9me3, H3K27me3, H3K27ac, Ring1b, CBX2, and EZH2 ChIP-seq data used in this study are available at ENCODE via accession codes ENCFF812HRW, ENCFF914VFE, ENCFF779QTH, ENCFF063UTI, ENCFF925XCF, ENCFF587SWK. The K562 whole genome bisulfite sequencing data used in this study are available at ENCODE via accession code ENCSR765JPC. The K562 DamID data used in this study are available at 4DNucleome via accession codes ENCSR765JPC and 4DNFIV776O7C. The K562 TRIP data used in this study are available at https://ocf.io/6qwj2/. The LAD sub-type definitions used in this study are available at https://osf.io/dk8pm/wiki/home/. CpG island genomic coordinates were obtained from UCSC (http://hgdownload.cse.ucsc.edu/goldenpath/hg38/database/cpgislandExt.txt.gz). The hg38 reference genome used for LIMe-Hi-C, ChIP-seq, and LIMe-ID alignment was obtained from NCBI (ftp://ftp.ncbi.nlm.nih.gov/genomes/all/GCA/000/001/405/GCA_000001405.15_GRCh38/seqs_for_alignment_pipelines.ucsc_ids/GCA_000001405.15_GRCh38_no_alt_analysis_set.fna.gz). The hg38 reference genome and annotation files for RNA-seq data analysis were obtained from Ensembl (ftp://ftp.ensembl.org/pub/release-101/fasta/homo_sapiens/dna/Homo_sapiens.GRCh38.dna.primary_assumbly/fa/gz) and (ftp://ftp.ensembl.org/pub/release-101/gtf/homo_sapiens/Homo_sapiens.GRCh38.101.gtf.gz). A full list of published data used in this study are detailed in Supplementary Table 1. LIMe LAD region calls are supplied in Supplementary Data 1. Sub-compartment calls are supplied in Supplementary Data 2. Regions gaining lamina attachment upon EZH2i are supplied in Supplementary Data 3. Differentially expressed genes are supplied in Supplementary Table 4. Source data are provided with this paper.

## Source data

Source Data

## Code availability

Code employed for analyzing the LIMe-Hi-C and LIMe-ID data is available upon request. Scripts for running per-read analysis are available in a Supplementary Software zip file.
