# Supplementary material for: Polycomb-lamina antagonism partitions heterochromatin at the nuclear periphery
Source: Nat Commun. 2022 Jul 20;13:4199. doi: 10.1038/s41467-022-31857-5 (PMC9300685; doi:10.1038/s41467-022-31857-5)
Supplement: Supplementary file 1 — Supplementary Information [file 41467_2022_31857_MOESM1_ESM.pdf]

Polycomb-lamina antagonism partitions heterochromatin at the nuclear periphery

Authors: Allison P. Siegenfeld[1,2,5], Shelby A. Roseman[1,2,5], Heejin Roh[1,2], Nicholas Z. Lue[1,2], Corin C. Wagen[1], Eric Zhou[1], Sarah E. Johnstone[2,3], Martin J. Aryee[2,4,*], Brian B. Liau[1,2,*]

Affiliations:
[1]Department of Chemistry and Chemical Biology, Harvard University, Cambridge, MA 02138, USA
[2]Broad Institute of Harvard and MIT, Cambridge, MA 02142, USA
[3]Department of Pathology, Dana-Farber Cancer Institute, Boston, MA 02115, USA
[4]Department of Data Science, Dana-Farber Cancer Institute, Boston, MA 02115, USA
[5]These authors contributed equally

***Correspondence:** martin.aryee@ds.dfci.harvard.edu; liau@chemistry.harvard.edu

This file includes:
Supplementary Table 1
Supplementary Figs. 1-7

**Analyzed Public Data**	**Source**	**Identifier**
K562 H3K9me3 ChIP-seq	Encode	ENCFF812HRW
K562 H3K27me3 ChIP-seq	Encode	ENCFF914VFE
K562 H3K27ac ChIP-seq	Encode	ENCFF779QTH
K562 Ring1b ChIP-seq	Encode	ENCFF063UTI
K562 CBX2 ChIP-seq	Encode	ENCFF925XCF
K562 EZH2 ChIP-seq	Encode	ENCFF587SWK
K562 Whole Genome Bisulfite Sequencing	Encode	ENCSR5765JPC
K562 Lamin B1 DamID Signal	4D Nucleome	4DNFIX4BXSIM
K562 Lamin B1 DamID Lamina Associated Domains (LADs)	4D Nucleome	4DNFIV776O7C
K562 TRIP data	Open Science Framework	https://osf.io/6qwj2/
KBM7 LAD sub-types	Open Science Framework	https://osf.io/dk8pm/wiki/home/

Supplementary Table 1 | Published datasets used in this study

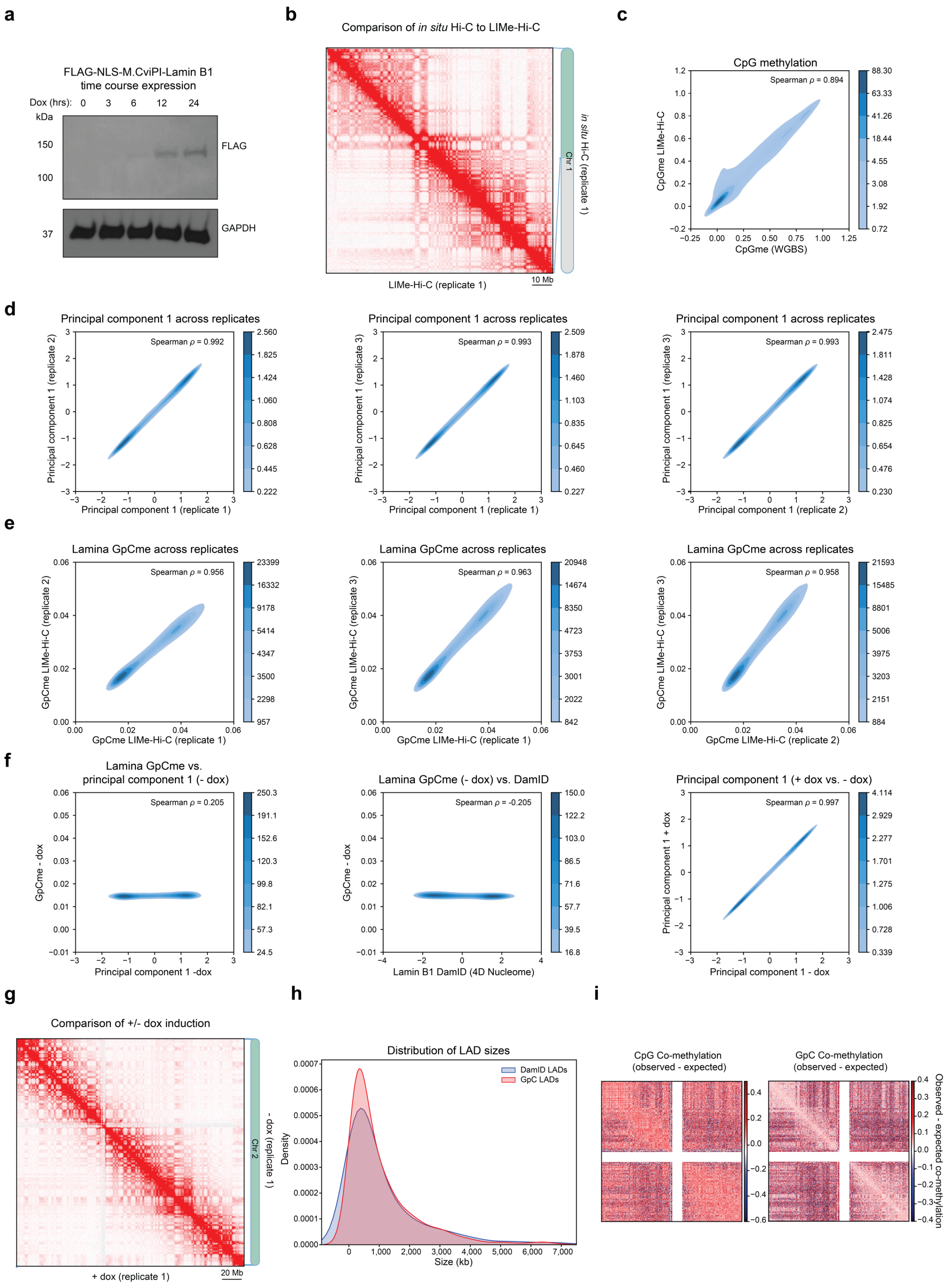
a
FLAG-NLS-M.CviPI-Lamin B1
time course expression
Dox (hrs): 0 3 6 12 24
kDa
150
100
37
FLAG
GAPDH
b
Comparison of in situ Hi-C to LIMe-Hi-C
in situ Hi-C (replicate 1)
Chr 1
LIMe-Hi-C (replicate 1)
10 Mb
c
CpG methylation
Spearman ρ = 0.894
CpGme LIMe-Hi-C
CpGme (WGBS)
d
Principal component 1 across replicates
Spearman ρ = 0.992
Spearman ρ = 0.993
Principal component 1 (replicate 1)
Principal component 1 (replicate 2)
Principal component 1 (replicate 3)
e
Lamina GpCme across replicates
Spearman ρ = 0.956
Spearman ρ = 0.963
Spearman ρ = 0.958
GpCme LIMe-Hi-C (replicate 1)
GpCme LIMe-Hi-C (replicate 2)
GpCme LIMe-Hi-C (replicate 3)
f
Lamina GpCme vs.
principal component 1 (- dox)
Spearman ρ = 0.205
GpCme - dox
Principal component 1 -dox
Lamina GpCme (- dox) vs. DamID
Spearman ρ = -0.205
Lamin B1 DamID (4D Nucleome)
Principal component 1 (+ dox vs. - dox)
Spearman ρ = 0.997
Principal component 1 + dox
Principal component 1 - dox
g
Comparison of +/- dox induction
- dox (replicate 1)
Chr 2
+ dox (replicate 1)
20 Mb
h
Distribution of LAD sizes
DamID LADs
GpC LADs
Density
Size (kb)
i
CpG Co-methylation
(observed - expected)
GpC Co-methylation
(observed - expected)
Observed - expected co-methylation

Supplementary Figure 1 | LIMe-Hi-C measurements of LADs, CpG methylation, and Hi-C contacts are robust

a) Immunoblot depicting levels of Flag-NLS-M.CviPI-Lamin B1 after specified amount of time of induction with doxycycline. GAPDH was utilized as a loading control. The experiment was performed once. Source data are provided as a Source Data file.
b) Hi-C contact map comparing LIMe-Hi-C (left) to *in situ* Hi-C (right) data for replicate 1 at 250 kb resolution for a region on chromosome 1.
c) Density heatmap comparing published whole genome bisulfite sequencing (WGBS) CpG methylation fraction (*x*-axis) to replicate-averaged LIMe-Hi-C CpG methylation fraction (*y*-axis) across 50 kb bins. Published WGBS dataset is specified in Supplementary Table 1.
d) Density heatmap comparing principal component 1 in an individual specified replicate (*x*-axis) to principal component 1 in a different specified replicate (*y*-axis) across 50 kb bins for + dox LIMe-Hi-C replicates.
e) Density heatmap comparing GpC methylation fraction in an individual specified replicate (*x*-axis) to GpC methylation fraction in a different specified replicate (*y*-axis) across 50 kb bins for + dox LIMe-Hi-C replicates.
f) Density heatmap comparing replicate-averaged GpC methylation fraction (*y*-axis) to replicate-averaged principal component 1 (*x*-axis) across 50 kb bins for the - dox LIMe-Hi-C condition (left). Density heatmap comparing replicate-averaged GpC methylation fraction (*y*-axis) for the - dox LIMe-Hi-C condition to published K562 DamID data (*x*-axis) across 50 kb bins (middle). Density heatmap comparing replicate-averaged principal component 1 across 50 kb bins for the + dox (*y*-axis) and - dox (*x*-axis) LIMe-Hi-C conditions (right). Published DamID dataset is specified in Supplementary Table 1.
g) Hi-C contact map comparing + dox (left) and - dox (right) conditions for replicate 1 at 250 kb resolution.
h) Density plot depicting the relative density (*y*-axis) of region sizes (*x*-axis) for LIMe and DamID LADs. The density function for each LAD type is independently scaled.
i) Observed - expected per-read CpG co-methylation (left) and GpC co-methylation (right) fraction for both arms of chromosome 1 (see Methods). Regions with low coverage near the centromere are marked in white.

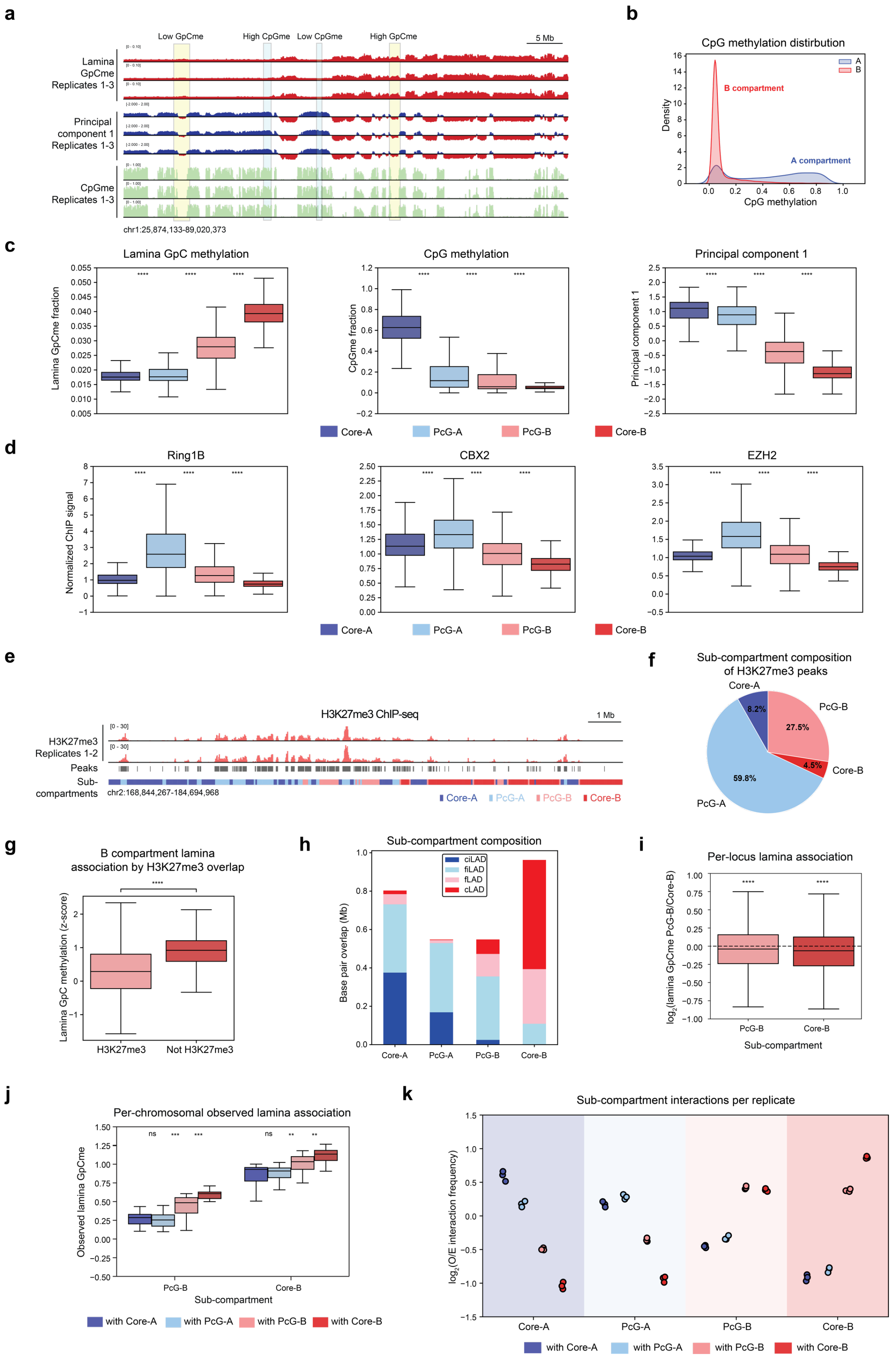
a
Low GpCme
High CpGme
Low CpGme
High GpCme
5 Mb
Lamina GpCme Replicates 1-3
Principal component 1 Replicates 1-3
CpGme Replicates 1-3
chr1:25,874,133-89,020,373
b
CpG methylation distirbution
B compartment
A compartment
Density
CpG methylation
c
Lamina GpC methylation
Lamina GpCme fraction
CpG methylation
CpGme fraction
Principal component 1
Principal component 1
Core-A
PcG-A
PcG-B
Core-B
d
Ring1B
Normalized ChIP signal
CBX2
EZH2
e
H3K27me3 ChIP-seq
1 Mb
H3K27me3 Replicates 1-2
Peaks
Sub-compartments
chr2:168,844,267-184,694,968
f
Sub-compartment composition of H3K27me3 peaks
8.2%
27.5%
4.5%
59.8%
g
B compartment lamina association by H3K27me3 overlap
Lamina GpC methylation (z-score)
H3K27me3
Not H3K27me3
h
Sub-compartment composition
ciLAD
fiLAD
fLAD
cLAD
Base pair overlap (Mb)
i
Per-locus lamina association
log2(lamina GpCme PcG-B/Core-B)
Sub-compartment
j
Per-chromosomal observed lamina association
Observed lamina GpCme
Sub-compartment
with Core-A
with PcG-A
with PcG-B
with Core-B
k
Sub-compartment interactions per replicate
log2(O/E interaction frequency)

Supplementary Figure 2 | Sub-compartments identified in K562 are epigenomically distinct

a) Genome browser tracks of lamina GpC methylation, principal component 1 and CpG methylation for LIMe-Hi-C replicates.
b) Density plot depicting the relative density (*y*-axis) of fraction CpG methylation (excluding CpG islands) (*x*-axis) for 50 kb bins across compartments. The density function for each compartment is independently scaled.
c) Boxplot of LIMe-Hi-C features (*y*-axis: fraction GpC methylation (left), fraction CpG methylation (middle), and principal component 1 (right)) across sub-compartments (*x*-axis).
d) Boxplot showing Polycomb factor enrichment (*y*-axis, fold-change over input/median signal) for 50 kb bins across sub-compartments (*x*-axis). Published datasets are specified in Supplementary Table 1.
e) Genome browser tracks of H3K27me3 ChIP-seq signal with H3K27me3 peaks and sub-compartments below.
f) Pie chart depicting fraction base pair overlap of sub-compartments with H3K27me3 peaks.
g) Boxplot of *z*-score-normalized lamina GpC methylation (*y*-axis) for 50 kb bins within the B compartment by their overlap with H3K27me3 peaks (*x*-axis).
h) Barplot depicting base pair overlap (*y*-axis) between sub-compartments (*x*-axis) and LAD sub-types. ciLAD: constitutive inter-LAD; fiLAD: facultative inter-LAD; fLAD: facultative LAD; cLAD: constitutive LAD.
i) Boxplot across 50 kb bins genome-wide for PcG-B and Core-B (*x*-axis) depicting the $\log_2$ ratio of the interval's lamina association status (*y*-axis) if it is interacting with the PcG-B versus Core-B. *P* values were calculated by a one-sample one-sided t-test to determine if the mean is less than 0.
j) Boxplot showing the normalized chromosome-wide average GpC methylation (*y*-axis) for loci as a function of the sub-compartment status of the loci's interaction partner for PcG-B and Core-B (*x*-axis) (see Methods).
k) Dotplot showing genome-wide average $\log_2$(observed/expected contact frequency) (*y*-axis) for every replicate across sub-compartments (*x*-axis). O/E denotes observed/expected.

In (**c**-**d**, **g**) *p* values were calculated by a Mann-Whitney-Wilcoxon two-sided test. In (**c**-**d**, **g, i-j**) the interquartile range (IQR) is depicted by the box with the median represented by the center line. Whiskers maximally extend to 1.5 × IQR (with outliers excluded). *P* values are annotated as follows: ns: not significant; *: $0.01<p\leq0.05$; **: $0.001<p\leq0.01$; ***: $0.0001<p\leq0.001$; ****: $p\leq0.0001$. Exact *p* values and the number of datapoints (n) compared are provided in the source data file.

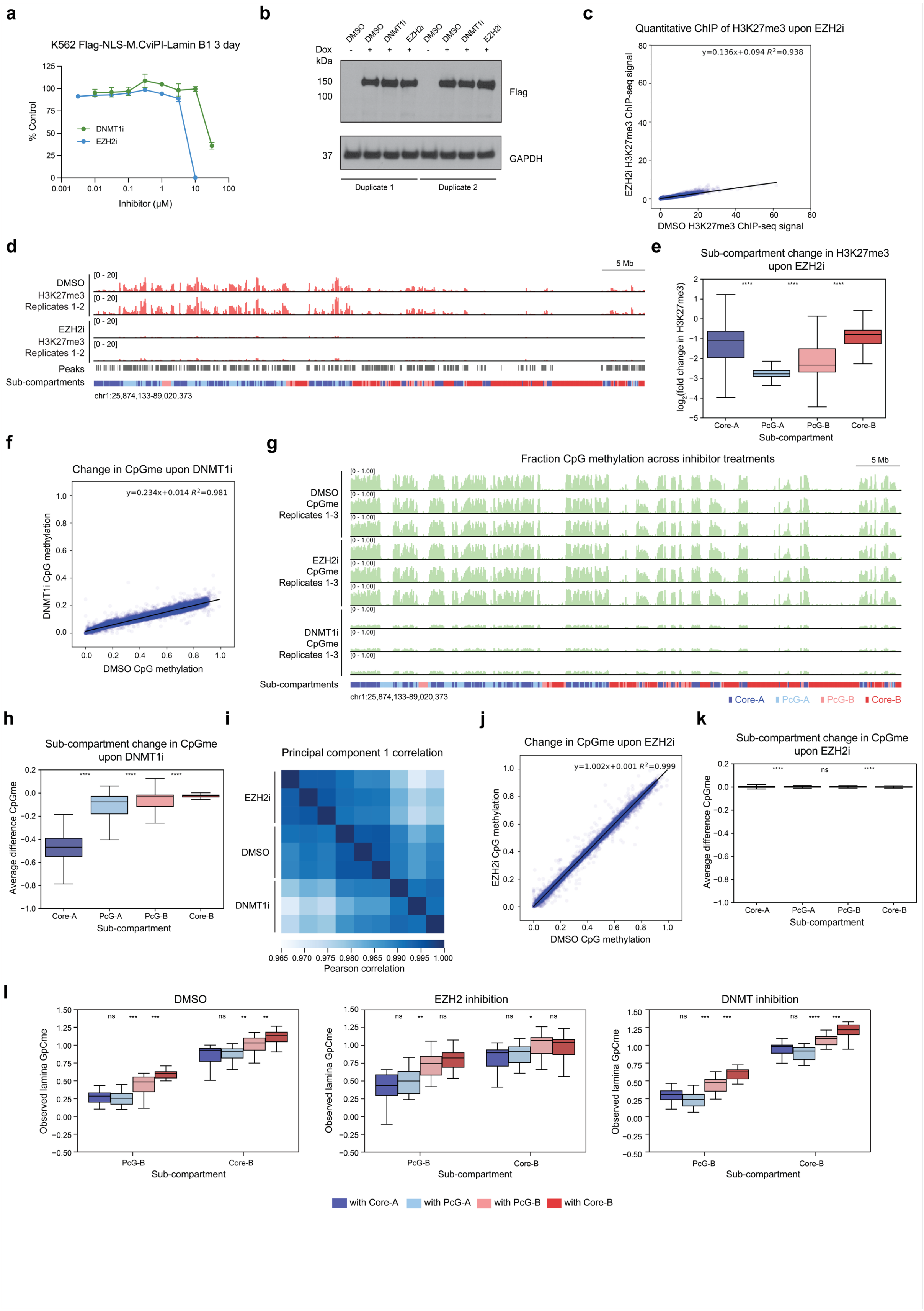
a
K562 Flag-NLS-M.CviPI-Lamin B1 3 day
% Control
DNMT1i
EZH2i
Inhibitor (μM)
b
DMSO
DNMT1i
EZH2i
Dox
kDa
150
100
37
Flag
GAPDH
Duplicate 1
Duplicate 2
c
Quantitative ChIP of H3K27me3 upon EZH2i
y=0.136x+0.094 R^2=0.938
EZH2i H3K27me3 ChIP-seq signal
DMSO H3K27me3 ChIP-seq signal
d
5 Mb
[0 - 20]
DMSO
H3K27me3
Replicates 1-2
EZH2i
H3K27me3
Replicates 1-2
Peaks
Sub-compartments
chr1:25,874,133-89,020,373
e
Sub-compartment change in H3K27me3
upon EZH2i
$\log_2$(fold change in H3K27me3)
Core-A
PcG-A
PcG-B
Core-B
Sub-compartment
f
Change in CpGme upon DNMT1i
y=0.234x+0.014 R^2=0.981
DNMT1i CpG methylation
DMSO CpG methylation
g
Fraction CpG methylation across inhibitor treatments
5 Mb
[0 - 1.00]
DMSO
CpGme
Replicates 1-3
EZH2i
CpGme
Replicates 1-3
DNMT1i
CpGme
Replicates 1-3
Sub-compartments
chr1:25,874,133-89,020,373
Core-A
PcG-A
PcG-B
Core-B
h
Sub-compartment change in CpGme
upon DNMT1i
Average difference CpGme
Sub-compartment
i
Principal component 1 correlation
EZH2i
DMSO
DNMT1i
0.965 0.970 0.975 0.980 0.985 0.990 0.995 1.000
Pearson correlation
j
Change in CpGme upon EZH2i
y=1.002x+0.001 R^2=0.999
EZH2i CpG methylation
DMSO CpG methylation
k
Sub-compartment change in CpGme
upon EZH2i
Average difference CpGme
Sub-compartment
l
DMSO
EZH2 inhibition
DNMT inhibition
Observed lamina GpCme
PcG-B
Core-B
Sub-compartment
with Core-A
with PcG-A
with PcG-B
with Core-B

Supplementary Figure 3 | Inhibition of EZH2 depletes H3K27me3 and inhibition of DNMT1 depletes DNA methylation globally

a) Dose-response curve of concentration inhibitor (*x*-axis) versus percent growth relative to vehicle (*y*-axis) for Flag-NLS-M.CviPI-Lamin B1 cells. Data represent mean ± s.e.m across three technical replicates with the experiment performed once. Source data are provided as a Source Data file.
b) Immunoblot for Flag-NLS-M.CviPI-Lamin B1 upon EZH2i, DNMT1i, and DMSO treatments +/- doxycycline. GAPDH was utilized as a loading control. The experiment was performed once in duplicate. Source data are provided as a Source Data file.
c) Scatterplot comparing replicate-averaged H3K27me3 ChIP-seq levels for DMSO (*x*-axis) and EZH2i (*y*-axis) treatments for 50 kb bins.
d) Genome browser tracks of H3K27me3 ChIP-seq signal for EZH2i and vehicle treatments.
e) Boxplot showing $\log_2$ fold-change H3K27me3 ChIP-seq signal between EZH2i and vehicle treatments for 50 kb bins (*y*-axis) across sub-compartments (*x*-axis).
f) Scatterplot comparing replicate-averaged CpG methylation fraction between DMSO (*x*-axis) and DNMT1i (*y*-axis) treatments for 50 kb bins.
g) Genome browser tracks of the CpG methylation fraction for LIMe-Hi-C samples.
h) Boxplot showing CpG methylation fraction change between DNMT1i and vehicle treatments for 50 kb bins (*y*-axis) across sub-compartments (*x*-axis).
i) Pearson correlation heatmap of principal component 1 across 50 kb bins for LIMe-Hi-C samples.
j) Scatterplot comparing replicate-averaged CpG methylation fraction between DMSO (*x*-axis) and EZH2i (*y*-axis) treatments for 50 kb bins.
k) Boxplot showing CpG methylation fraction change between EZH2i and vehicle treatments for 50 kb bins (*y*-axis) across sub-compartments (*x*-axis).
l) Boxplots showing the normalized chromosome-wide average GpC methylation (*y*-axis) for loci as a function of the sub-compartment status of the loci's interaction partner for PcG-B and Core-B regions (*x*-axis) across treatments (see Methods).

In (**e**, **h**, **k-l**) the interquartile range (IQR) is depicted by the box with the median represented by the center line. Whiskers maximally extend to 1.5 × IQR (with outliers excluded). *P* values were calculated by a Mann-Whitney-Wilcoxon two-sided test and are annotated as follows: ns: not significant; *: $0.01<p\leq0.05$; **: $0.001<p\leq0.01$; ***: $0.0001<p\leq0.001$; ****: $p\leq0.0001$. Exact *p* values and the number of datapoints (n) compared are provided in the source data file. In (**c**, **f**, **j**), the line of best fit is depicted.

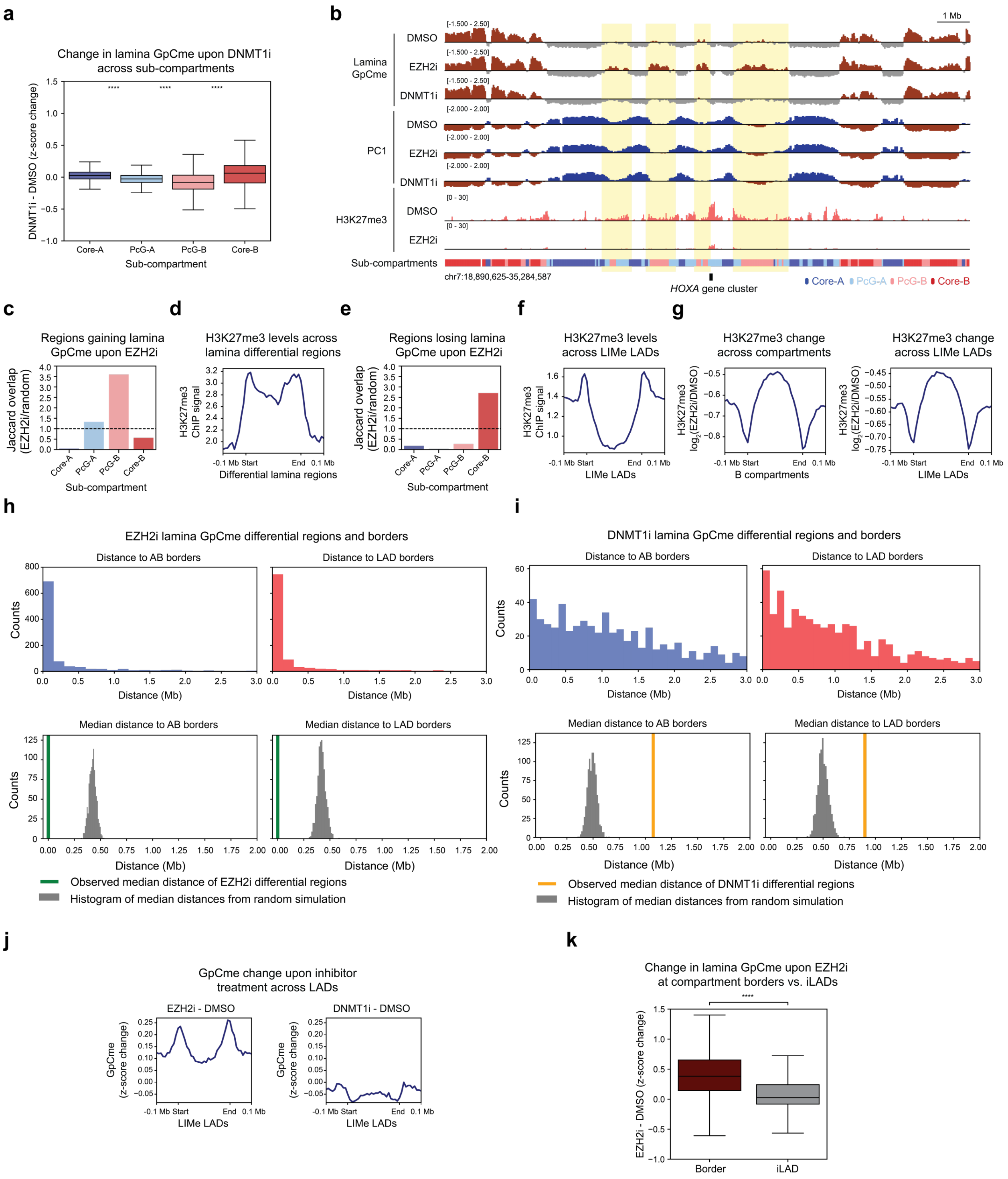

Supplementary Figure 4 | Regions gaining lamina association upon EZH2 inhibition are enriched within PcG-B and reside at LAD and compartment borders

a) Boxplot showing *z*-score-normalized GpC methylation change between DNMT1i and vehicle treatments for 50 kb bins (*y*-axis) across sub-compartments (*x*-axis).

b) Genome browser tracks of replicate-averaged *z*-score-normalized lamina GpC methylation levels, principal component 1, and H3K27me3 ChIP-seq signal for LIMe-Hi-C and ChIP-seq data near the *HOXA* locus.
c) Barplot showing for every sub-compartment (*x*-axis) the observed/simulated Jaccard overlap (*y*-axis) for regions gaining lamina contact upon EZH2 inhibition relative to a simulated random distribution.
d) Aggregate profile plot of H3K27me3 ChIP-seq signal for vehicle treatment (*y*-axis) across regions gaining lamina contact upon EZH2 inhibition (*x*-axis) (see Methods).
e) Barplot showing for every sub-compartment (*x*-axis) the observed/simulated Jaccard overlap (*y*-axis) for regions losing lamina contact upon EZH2 relative to a simulated random distribution.
f) Aggregate profile plot of H3K27me3 ChIP-seq signal for vehicle treatment (*y*-axis) across LIMe LADs (*x-axis*).
g) Aggregate profile plot of $\log_2$ fold-change H3K27me3 ChIP-seq signal between EZH2 inhibition and vehicle treatments (*y*-axis) across B compartments (left, *x*-axis) and LIMe LADs (right, *x*-axis).
h) Histogram of the frequency (*y*-axis) of distances (*x*-axis) for regions gaining lamina contact upon EZH2 inhibition to A/B or LAD borders (top). Histogram of the observed counts (y-axis) of median distances (*x*-axis) of randomly shuffled regions to A/B or LAD borders (bottom).
i) Histogram as in **h** for regions gaining lamina contact upon DNMT1 inhibition.
j) Aggregate profile plots depicting change in *z*-score-normalized lamina GpC methylation (*y*-axis) across LIMe LADs (*x*-axis) upon inhibitor treatments.
k) Boxplot showing the difference in *z*-score-normalized GpC methylation between EZH2i and vehicle treatments (*y*-axis) for H3K27me3 peaks within 50 kb of compartment borders versus those further than 50 kb away within the A compartment (*x*-axis).

In (**a**, **k**) the interquartile range (IQR) is depicted by the box with the median represented by the center line. Whiskers maximally extend to 1.5 × IQR (with outliers excluded). *P* values were calculated by a Mann-Whitney-Wilcoxon two-sided test and are annotated as follows: ns: not significant; *: $0.01<p\leq0.05$; **: $0.001<p\leq0.01$; ***: $0.0001<p\leq0.001$; ****: $p\leq0.0001$. Exact *p* values and the number of datapoints (n) compared are provided in the source data file.

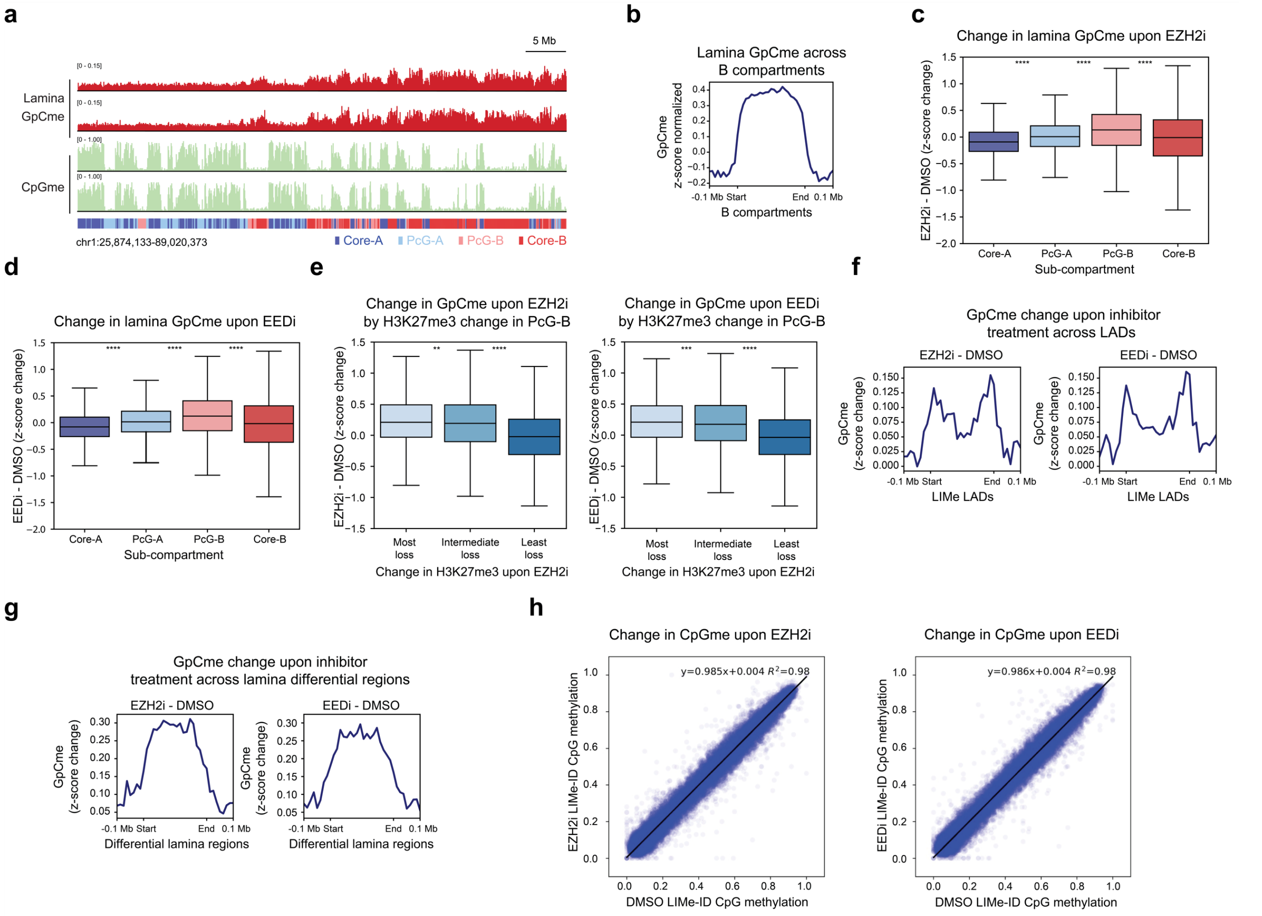

Supplementary Figure 5 | LIMe-ID can be used to profile lamina association and reveals that EED inhibition recapitulates the effects of EZH2 inhibition

a) Genome browser tracks of GpC and CpG methylation fraction for vehicle treatment for LIMe-ID duplicates with sub-compartment designation below.
b) Aggregate profile plot of LIMe-ID replicate-averaged *z*-score-normalized GpC methylation (*y*-axis) across B compartments (*x*-axis).
c) Boxplot showing *z*-score-normalized GpC methylation change between inhibitor and vehicle treatment for 50 kb bins (*y*-axis) across sub-compartments (*x*-axis) for the EZH2 inhibitor LIMe-ID data.
d) Boxplot showing *z*-score-normalized GpC methylation change between inhibitor and vehicle treatment for 50 kb bins (*y*-axis) across sub-compartments (*x*-axis) for the EED inhibitor LIMe-ID data.
e) Boxplots showing the difference in *z*-score-normalized GpC methylation between inhibitor and vehicle treatment for 50 kb bins (*y*-axis) segregated into three equally-sized quantiles by $\log_2$ fold-change in H3K27me3 (*x*-axis) for PcG-B domains for both EZH2 inhibitor LIMe-ID data (left) and EED inhibitor LIMe-ID data (right).
f) Aggregate profile plots depicting change in *z*-score-normalized lamina GpC methylation (*y*-axis) across LIMe LADs (*x*-axis) upon EZH2 inhibition (left) and EED inhibition (right) (see Methods).

g) Aggregate profile plots depicting change in *z*-score-normalized lamina GpC methylation (*y*-axis) across EZH2 LIMe-Hi-C lamina differential regions (*x*-axis) for EZH2 inhibition (left) and EED inhibition (right) (see Methods).
h) Scatterplot comparing CpG methylation fraction between DMSO (*x*-axis) and EZH2 inhibitor treatment (left, *y*-axis) and EED inhibitor treatment (right, *y*-axis) for 50 kb bins averaged across replicates. Equation for the line of best fit is depicted in the plot.

In (**c**-**e**) the interquartile range (IQR) is depicted by the box with the median represented by the center line. Whiskers maximally extend to 1.5 × IQR (with outliers excluded). *P* values were calculated by a Mann-Whitney-Wilcoxon two-sided test and are annotated as follows: ns: not significant; *: $0.01<p\leq0.05$; **: $0.001<p\leq0.01$; ***: $0.0001<p\leq0.001$; ****: $p\leq0.0001$. Exact *p* values and the number of datapoints (n) compared are provided in the source data file.

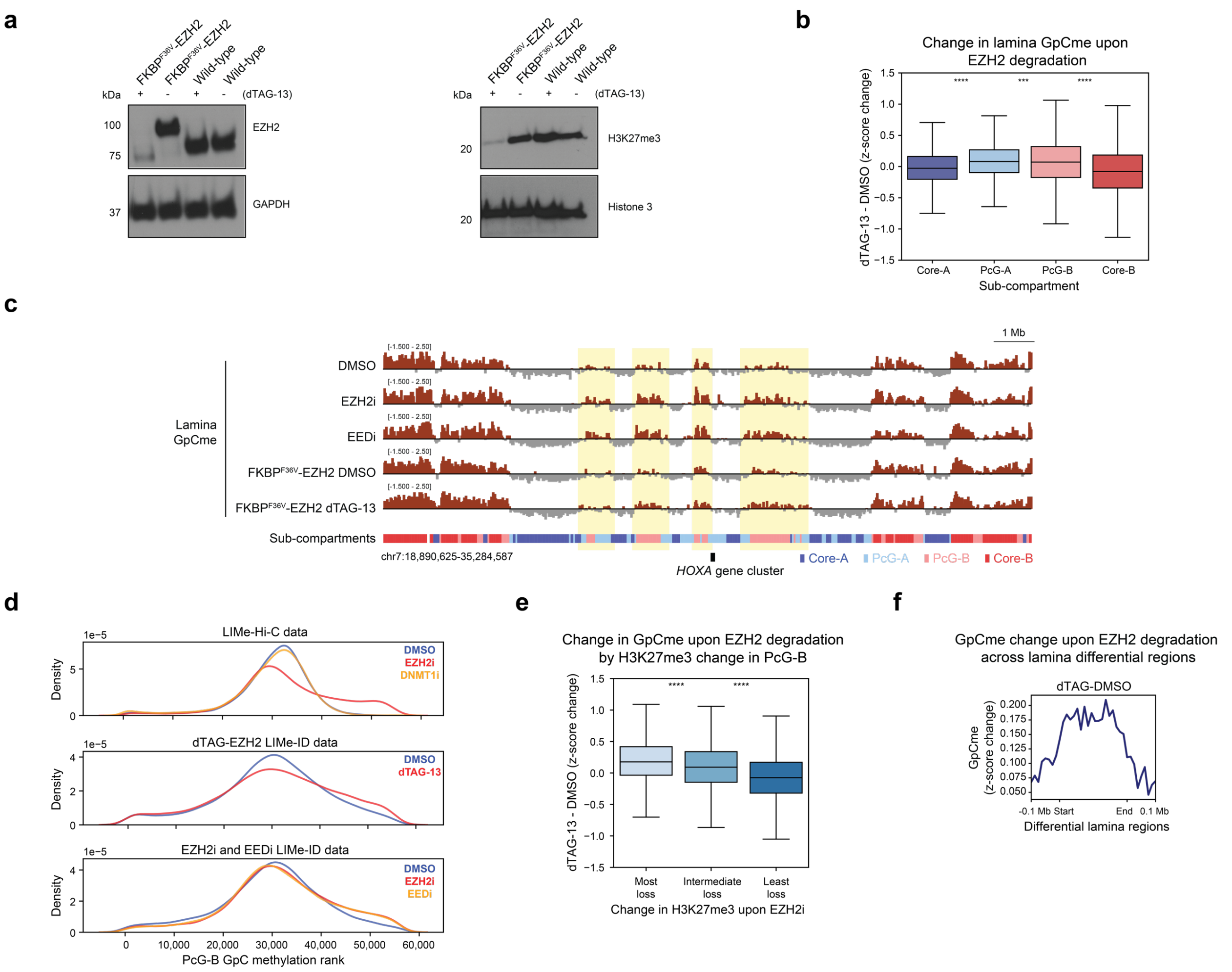

Supplementary Figure 6 | LIMe-ID following EZH2 degradation supports the role of H3K27me3 in antagonizing lamina association

a) Immunoblot depicting levels of EZH2 for the N-terminal EZH2 knock-in cell line and wild-type K562 upon DMSO and 72 hours dTAG-13 treatment. GAPDH was utilized as a loading control (left). Histone immunoblot depicting levels of H3K27me3 for the N-terminal EZH2 knock-in cell line and wild-type K562 upon DMSO and dTAG-13 treatment. Histone 3 was utilized as a loading control (right). The experiment was performed once. Source data are provided as a Source Data file.

b) Boxplot showing *z*-score-normalized GpC methylation change between dTAG-13 and vehicle treatment for 50 kb bins (*y*-axis) across sub-compartments (*x*-axis).

c) Genome browser tracks of *z*-score-normalized lamina GpC methylation levels averaged across replicates for LIMe-ID inhibitor treatments and EZH2 degradation near the *HOXA* locus.

d) Density plot of GpC methylation rank for PcG-B regions for the LIMe-Hi-C inhibitor treatments as well as the LIMe-ID inhibitor treatments and EZH2 degradation.

e) Boxplot showing the difference in *z*-score-normalized GpC methylation between dTAG-13 and vehicle treatment for 50 kb bins (*y*-axis) segregated into three equally-sized quantiles by $\log_2$ fold-change in H3K27me3 (*x*-axis) for PcG-B.

f) Aggregate profile plot depicting change in *z*-score-normalized lamina GpC methylation (*y*-axis) across EZH2 LIMe-Hi-C lamina differential regions (*x*-axis) for dTAG-13 treatment (see Methods).

In (**b**, **e**) the interquartile range (IQR) is depicted by the box with the median represented by the center line. Whiskers maximally extend to 1.5 × IQR (with outliers excluded). *P* values were calculated by a Mann-Whitney-Wilcoxon two-sided test and are annotated as follows: ns: not significant; *: 0.01<p≤0.05; **: 0.001<p≤0.01; ***: 0.0001<p≤0.001; ****: p≤0.0001. Exact *p* values and the number of datapoints (n) compared are provided in the source data file.

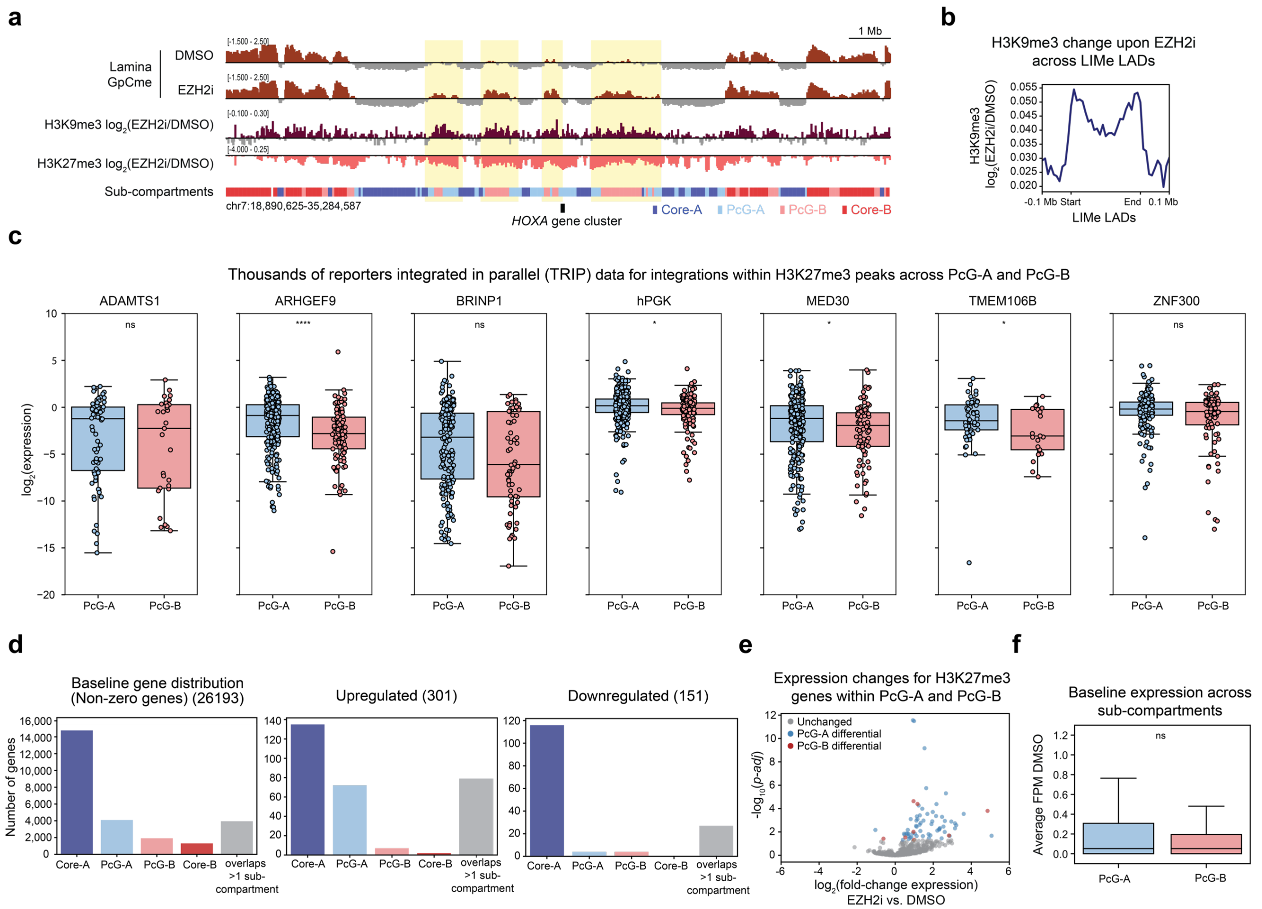

Supplementary Figure 7 | Polycomb sub-compartments represent distinct transcriptional environments

a) Genome browser tracks of *z*-score-normalized lamina GpC methylation levels averaged across replicates for the LIMe-Hi-C data, $\log_2$ fold-change H3K9me3 ChIP-seq, and $\log_2$ fold-change H3K27me3 ChIP-seq signal between EZH2i and vehicle treatment near the *HOXA* locus.
b) Aggregate profile plot of $\log_2$ fold-change H3K9me3 ChIP-seq signal between EZH2 inhibition and vehicle treatment (*y*-axis) across LIMe LADs (*x*-axis).
c) Boxplot with individual points overlaid of $\log_2$(TRIP expression) (*y*-axis) across the PcG-A and PcG-B sub-compartments (*x*-axis) for promoter integrations that overlap with H3K27me3 peaks.
d) Bar charts depicting number of genes within each sub-compartment for those that are detected by RNA-seq (non-zero) and those that are either upregulated or downregulated upon EZH2 inhibition.
e) Volcano plot depicting $-\log_{10}$(adjusted *p*-value) (*y*-axis) relative to $\log_2$(fold-change expression) (*x*-axis) between EZH2 inhibition and vehicle treatment for H3K27me3-positive PcG-A and PcG-B genes. *P* values were calculated by the Wald test and corrected for multiple testing using the Benjamini-Hochberg method.
f) Boxplot of baseline expression (*y*-axis, fragment per million mapped fragments (FPM)) for vehicle treatment of non-zero genes that overlap with H3K27me3 peaks across PcG-A and PcG-B (*x*-axis). Outlier points are excluded.

In (**c**, **f**) the interquartile range (IQR) is depicted by the box with the median represented by the center line. Whiskers maximally extend to 1.5 × IQR. *P* values were calculated by a Mann-

Whitney-Wilcoxon two-sided test and are annotated as follows: ns: not significant; *: $0.01<p\leq0.05$; **: $0.001<p\leq0.01$; ***: $0.0001<p\leq0.001$; ****: $p\leq0.0001$. Exact *p* values and the number of datapoints (n) compared are provided in the source data file.
