# Supplementary material for: Polycomb-lamina antagonism partitions heterochromatin at the nuclear periphery
Source: Nat Commun. 2022 Jul 20;13:4199. doi: 10.1038/s41467-022-31857-5 (PMC9300685; doi:10.1038/s41467-022-31857-5)
Supplement: Supplementary file 2 — Description of additional Supplementary File [file 41467_2022_31857_MOESM2_ESM.pdf]

Descriptions of Additional Supplementary Data files

Supplementary Data 1. LIMe-LAD coordinates. The chromosome, start position and end position for all LADs identified by LIMe-Hi-C are specified.

Supplementary Data 2. Sub-compartment coordinates. For everyone 50 kb bin the chromosome, start position and end position are specified along with the sub-compartment designation. Bins with no sub-compartment identifier are represented by ".".

Supplementary Data 3. Regions gaining lamina contact following EZH2 inhibition. The chromosome, start position and end position for all regsions identified by LIMe-Hi-C to be gaining lamina association following EZH2 inhibition are specified.

Supplementary Data 4. Differentially expressed genes following EZH2 inhibition. The Ensembl identifier (ENSG), average normalized expression across samples (baseMean), log2 fold-change in expression (log2FoldChange), log2 fold change standard error (lfcSE), Wald statistic (stat), p-value calculated by a Wald test, and adjusted p-value are supplied for all differentially expressed genes.
