# Supplementary material for: Polycomb-lamina antagonism partitions heterochromatin at the nuclear periphery
Source: Nat Commun. 2022 Jul 20;13:4199. doi: 10.1038/s41467-022-31857-5 (PMC9300685; doi:10.1038/s41467-022-31857-5)
Supplement: Supplementary file 8 — Reporting Summary [file 41467_2022_31857_MOESM8_ESM.pdf]

nature portfolio

Corresponding author(s): Brian B. Liau, Martin J. Aryee

Last updated by author(s): 2022/06/17

Reporting Summary

Nature Portfolio wishes to improve the reproducibility of the work that we publish. This form provides structure for consistency and transparency in reporting. For further information on Nature Portfolio policies, see our Editorial Policies and the Editorial Policy Checklist.

Statistics

For all statistical analyses, confirm that the following items are present in the figure legend, table legend, main text, or Methods section.

n/a	Confirmed	
☐	☒	The exact sample size (*n*) for each experimental group/condition, given as a discrete number and unit of measurement
☐	☒	A statement on whether measurements were taken from distinct samples or whether the same sample was measured repeatedly
☐	☒	The statistical test(s) used AND whether they are one- or two-sided<br>*Only common tests should be described solely by name; describe more complex techniques in the Methods section.*
☒	☐	A description of all covariates tested
☐	☒	A description of any assumptions or corrections, such as tests of normality and adjustment for multiple comparisons
☐	☒	A full description of the statistical parameters including central tendency (e.g. means) or other basic estimates (e.g. regression coefficient) AND variation (e.g. standard deviation) or associated estimates of uncertainty (e.g. confidence intervals)
☐	☒	For null hypothesis testing, the test statistic (e.g. *F*, *t*, *r*) with confidence intervals, effect sizes, degrees of freedom and *P* value noted<br>*Give P values as exact values whenever suitable.*
☒	☐	For Bayesian analysis, information on the choice of priors and Markov chain Monte Carlo settings
☒	☐	For hierarchical and complex designs, identification of the appropriate level for tests and full reporting of outcomes
☐	☒	Estimates of effect sizes (e.g. Cohen's *d*, Pearson's *r*), indicating how they were calculated

Our web collection on statistics for biologists contains articles on many of the points above.

Software and code

Policy information about availability of computer code

Data collection	RNA-seq, ChIP-seq, LIMe-Hi-C, LIMe-ID and Hi-C experiments were sequenced on an Illumina Novaseq (SP or S4) instrument.<br><br>Luminescence measurements for the growth assays were taken on the the SpectraMax i3x microplate reader (Molecular Devices) with the SoftMax Pro (version 6.5.1) software.
Data analysis	The LIMe-Hi-C data were aligned and processed utilizing the CPU version of the JuiceMe (version 1.0.0) with bwa-meth (version 0.2.2) and Juicertools (version 1.22.01). In situ Hi-C data were processed according to the SLURM version of the Juicer pipeline (version 1.6) with Juicertools (version 1.22.01). The Biscuit pileup program (version 0.3.16) was utilized to assess LIMe-Hi-C DNA methylation. The UCSC bedGraphToBigWig function (version 4) was used to obtain methylation bigwig files. Homer (version 4.10) was utilized for principal component 1 analysis. Differential GpC methylation and LIMe-LADs were determined in R (version 3.6.1) with Bumphunter (version 1.34.0). Metaprofile plots and bin-level averages were generated using deepTools (version 3.4.3). Sub-compartment clusters were identified in R (version 3.6.1) using the pheatmap clustering and plotting function (version 1.0.12). Saddle plot analysis was performed with cooltools (version 0.4.0) using contact files converted to the cool format with the hic2cool function (version 0.8.3). Pybedtools (version 0.8.1) and Bedtools2 (version 2.26.0) were utilized to manipulate genomic intervals as well as assess their overlap.<br><br>LIMe-ID analysis was performed analogously to the LIMe-Hi-C analysis except that reads were aligned utilizing the Biscuit alignment pipeline with the following parameters: “biscuit align -t 16 -R '@RG\tID' Read1.fastq.gz Read2fastq.gz | samblaster | samtools sort -o sample.bam -O BAM –” (Biscuit, version 0.3.16). The Biscuit vcf2bed program was employed with the parameters “-k 1” instead of “-k 3” to account for the lower sequencing depth utilized for LIMe-ID (Biscuit, version 0.3.16).<br><br>ChIP-seq data was analyzed with the Spiker analysis framework (version 1.03) with samtools (version 0.1.19) and deepTools (version 3.4.3). MACS2 (version 2.1.1.20160309) was utilized to call ChIP-seq peaks. See the ChIP-seq section for more details.

Data visualization and processing of the LIMe-Hi-C, LIMe-ID, and ChIP-seq data were performed in Python (version 3.7.0) and R (version 3.6.1) with Scipy (version 1.2.1), seaborn (version 0.11.1), and matplotlib (version 3.0.3). Genome browser tracks and Hi-C heatmaps were visualized using the Integrated Genome Browser (version 2.5.3) and Juicebox (version 1.11.08) software.

RNA-seq count files were generated using HTSeq (version 0.11.2) and was subsequently processed in R (version 4.0.2) with the following packages: DESeq2 (version 1.28.1), and BioMart (version 2.44.1). Bedtools2 (version 2.26.) was utilized to assess overlap of genes with H3K27me3 ChIP-seq peaks.

Code for the per-read multi-modal analysis is provided in a Supplementary Zip file.

For manuscripts utilizing custom algorithms or software that are central to the research but not yet described in published literature, software must be made available to editors and reviewers. We strongly encourage code deposition in a community repository (e.g. GitHub). See the Nature Portfolio guidelines for submitting code & software for further information.

Data

Policy information about availability of data

All manuscripts must include a data availability statement. This statement should provide the following information, where applicable:

- Accession codes, unique identifiers, or web links for publicly available datasets
- A description of any restrictions on data availability
- For clinical datasets or third party data, please ensure that the statement adheres to our policy

All sequencing data has been deposited on NCBI Gene Expression Omnibus under the super series accession code GSE180230 [https://www.ncbi.nlm.nih.gov/geo/query/acc.cgi?acc=GSE180230]. The LIMe-Hi-C data generated in this study have been deposited in the NCBI Gene Expression Omnibus under accession code GSE180228 [https://www.ncbi.nlm.nih.gov/geo/query/acc.cgi?acc=GSE180228]. The RNA-seq data generated in this study have been deposited in the NCBI Gene Expression Omnibus under accession code GSE180229 [https://www.ncbi.nlm.nih.gov/geo/query/acc.cgi?acc=GSE180229]. The ChIP-seq data generated in this study have been deposited in the NCBI Gene Expression Omnibus under accession code GSE198613 [https://www.ncbi.nlm.nih.gov/geo/query/acc.cgi?acc=GSE198613]. The LIMe-ID data generated in this study have been deposited in the NCBI Gene Expression Omnibus under accession code GSE198615. The K562 H3K9me3, H3K27me3, H3K27ac, Ring1b, CBX2, and EZH2 ChIP-seq data used in this study are available at [www.encodeproject.org/files/ENCFF812HRW], [www.encodeproject.org/files/ENCFF914VFE], [www.encodeproject.org/files/ENCFF779QTH], [www.encodeproject.org/files/ENCFF063UTI], [www.encodeproject.org/files/ENCFF925XCF], [www.encodeproject.org/files/ENCFF587SWK]. The K562 whole genome bisulfite sequencing data used in this study are available at [www.encodeproject.org/experiments/ENCSR765JPC]. The K562 DamID data used in this study are available at [data.4dnucleome.org/files-processed/4DNFIX4BXSIM/] and [data.4dnucleome.org/files-processed/4DNFIV776O7C/]. The K562 TRIP data used in this study are available at [https://ocf.io/6qwj2/]. The LAD sub-type definitions used in this study are available at https://osf.io/dk8pm/wiki/home/. CpG island genomic coordinates were obtained from UCSC [http://hgdownload.cse.ucsc.edu/goldenpath/hg38/database/cpgislandExt.txt.gz]. The hg38 reference genome used for LIMe-Hi-C, ChIP-seq, and LIMe-ID alignment was obtained from NCBI [ftp://ftp.ncbi.nlm.nih.gov/genomes/all/GCA/000/001/405/GCA_000001405.15_GRCh38/seqs_for_alignment_pipelines.ucsc_ids/GCA_000001405.15_GRCh38_no_alt_analysis_set.fna.gz]. The hg38 reference genome and annotation files for RNA-seq data analysis were obtained from Ensembl [ftp://ftp.ensembl.org/pub/release-101/fasta/homo_sapiens/dna/Homo_sapiens.GRCh38.dna.primary_assumbly/fa/gz] and [ftp://ftp.ensembl.org/pub/release-101/gtf/homo_sapiens/Homo_sapiens.GRCh38.101.gtf.gz] A full list of published data used in this study are detailed in Supplementary Table 1. LIMe LAD region calls are supplied in Supplementary Data 1. Sub-compartment calls are supplied in Supplementary Data 2. Regions gaining lamina attachment upon EZH2i are supplied in Supplementary Data 3. Differentially expressed genes are supplied in Supplementary Table 4. Source data are provided with this paper.

Field-specific reporting

Please select the one below that is the best fit for your research. If you are not sure, read the appropriate sections before making your selection.

☒ Life sciences ☐ Behavioural & social sciences ☐ Ecological, evolutionary & environmental sciences

For a reference copy of the document with all sections, see nature.com/documents/nr-reporting-summary-flat.pdf

Life sciences study design

All studies must disclose on these points even when the disclosure is negative.

Sample size	ChIP-seq experiments were performed in duplicate and RNA-seq experiments were performed in triplicate, following standards published by the ENCODE consortium. Consistent with published Hi-C datasets and the ENCODE consortium's standards for whole genome bisulfite sequencing, three replicates were performed for LIMe-Hi-C. This was found to be sufficient based on the quality control analysis for GpC methylation and principal component 1 across replicates in Figure S1. LIMe-ID experiments were performed in duplicate, also consistent with ENCODE consortium standards for whole genome bisulfite sequencing and previous DamID studies.
Data exclusions	For the sub-compartment analysis, 50 kb regions that in the DMSO + dox condition had a replicate-average |z-score value| > 3 for CpGme, GpCme or principal component 1 were excluded from the classification and analysis as they were deemed to be outliers. These bins were excluded from subsequent analyses.<br><br>For the RNA-seq analysis transcripts with zero reads were excluded.
Replication	Genomics experiments were performed with multiple biological replicates, detailed above. Growth assays were performed once with three technical replicates. Immunoblots were performed in once.
Randomization	No randomization was performed for practical reasons of ensuring that each sample was subjected to the correct treatment condition.

However, we did ensure that there were equal number of treatment and control samples for every replicate. In addition, all samples for a given experiment were processed at the same time to control for batch effects.

Blinding

None of the experiments performed in this study involved the blinding of investigators. Investigators were not blinded for practical purposes of running the experiments, as it was essential to distinguish which conditions were drug versus vehicle treated. Blinding was also unnecessary since comparisons were made using statistical software where all samples were processed using identical pipelines and consequently not influenced by the investigator.

Reporting for specific materials, systems and methods

We require information from authors about some types of materials, experimental systems and methods used in many studies. Here, indicate whether each material, system or method listed is relevant to your study. If you are not sure if a list item applies to your research, read the appropriate section before selecting a response.

Materials & experimental systems

n/a	Involved in the study
☐	☒ Antibodies
☐	☒ Eukaryotic cell lines
☒	☐ Palaeontology and archaeology
☒	☐ Animals and other organisms
☒	☐ Human research participants
☒	☐ Clinical data
☒	☐ Dual use research of concern

Methods

n/a	Involved in the study
☐	☒ ChIP-seq
☒	☐ Flow cytometry
☒	☐ MRI-based neuroimaging

Antibodies

Antibodies used

The H3K27me3 antibody (Cell Signaling Technology, C36B11 cat# 9733S Lot #19, 1:80) and the H3K9me3 antibody (Cell Signaling, cat# 13969S Lot #3, 1:75) along with the spike-in antibody (Active Motif, cat# 61686, 0.4ug/million cells) were utilized for quantitative ChIP-seq. The primary antibodies used for immunoblotting are as follows: GAPDH (Santa Cruz Biotechnology cat# sc-477724, 1:5000); monoclonal anti-FLAG M2 (Sigma-Aldrich cat #F1804, 1:1000 and 1:5000); Tri-Methyl-Histone H3 Lys27 (Cell Signaling Technology C36B11, cat# 9733S, 1:10,000); Histone H3 (Cell Signaling Technology cat# 9715S, 1:10,000) and EZH2 (Cell Signaling Technology cat# 5246S, 1:4000).

Validation

The H3K27me3 and H3K9me3 antibodies (Cell Signaling Technology, cat# 9733S and 13969S) have been validated for ChIP-seq using SimpleChIP® Enzymatic Chromatin IP Kits, as stated on the manufacturer's website. The GAPDH (Santa Cruz Biotechnology cat# sc-477724), anti-FLAG M2 (Sigma-Aldrich cat #F1804), Tri-Methyl-Histone H3 Lys27 (Cell Signaling cat# 9733S), Histone H3 (Cell Signaling Technology cat# 9715S), and EZH2(Cell Signaling Technology cat# 5246S) antibodies have all been validated for western blotting as stated on the manufacturer's websites. The spike-in antibody (Active Motif, cat# 61686) has been validated for use in spike-in ChIP-seq and reacts with a drosophila-specific histone variant. All other antibodies react with human targets.

Eukaryotic cell lines

Policy information about cell lines

Cell line source(s)

K562 (ATCC). HEK 293T (Bradley E. Bernstein, Massachusetts General Hospital - originally Life Technologies).

Authentication

K562 were authenticated with short tandem repeat (STR) profiling through Sigma Aldrich. HEK 293T cells were not authenticated.

Mycoplasma contamination

Cell lines were tested routinely throughout the duration of the study for mycoplasma. In each case, all cell lines tested negative.

Commonly misidentified lines
(See ICLAC register)

No commonly misidentified lines were used in this study.

ChIP-seq

Data deposition

☒ Confirm that both raw and final processed data have been deposited in a public database such as GEO.

☒ Confirm that you have deposited or provided access to graph files (e.g. BED files) for the called peaks.

Data access links
May remain private before publication.

The data has been uploaded to GEO under the superseries GSE180230.

Files in database submission

K562_DMSO_H3K27me3_rep1.bw
K562_DMSO_H3K27me3_rep2.bw

K562_DMSO_H3K9Me3_rep1.bw
K562_DMSO_H3K9Me3_rep2.bw
K562_GSK343_H3K27me3_rep1.bw
K562_GSK343_H3K27me3_rep2.bw
K562_GSK343_H3K9Me3_rep1.bw
K562_GSK343_H3K9Me3_rep2.bw
K562_DMSO_H3K27me3_rep1_R1.fastq.gz
K562_DMSO_H3K27me3_rep1_R2.fastq.gz
K562_DMSO_H3K27me3_rep2_R1.fastq.gz
K562_DMSO_H3K27me3_rep2_R2.fastq.gz
K562_DMSO_H3K9me3_rep1_R1.fastq.gz
K562_DMSO_H3K9me3_rep1_R2.fastq.gz
K562_DMSO_H3K9me3_rep2_R1.fastq.gz
K562_DMSO_H3K9me3_rep2_R2.fastq.gz
K562_GSK343_H3K27me3_rep1_R1.fastq.gz
K562_GSK343_H3K27me3_rep1_R2.fastq.gz
K562_GSK343_H3K27me3_rep2_R1.fastq.gz
K562_GSK343_H3K27me3_rep2_R2.fastq.gz
K562_GSK343_H3K9me3_rep1_R1.fastq.gz
K562_GSK343_H3K9me3_rep1_R2.fastq.gz
K562_GSK343_H3K9me3_rep2_R1.fastq.gz
K562_GSK343_H3K9me3_rep2_R2.fastq.gz
K562_GSK343_input_rep1_R1.fastq.gz
K562_GSK343_input_rep1_R2.fastq.gz
K562_DMSO_input_rep1_R1.fastq.gz
K562_DMSO_input_rep1_R2.fastq.gz
K562_GSK343_input_rep2_R1.fastq.gz
K562_GSK343_input_rep2_R2.fastq.gz
K562_DMSO_input_rep2_R1.fastq.gz
K562_DMSO_input_rep2_R2.fastq.gz
H3K27me3_DMSO_rep1_rep2_peaks.bed

Genome browser session (e.g. UCSC)

no longer applicable

Methodology

Replicates

Experiments were performed in duplicate.

Sequencing depth

About 20,000,000 paired-end reads of 50 bp length were obtained per sample. Duplication rate was assessed upon peak calling and found to be <1%.

Antibodies

The H3K27me3 antibody (Cell Signaling, C36B11 Lot #19) and the H3K9me3 antibody (Cell Signaling, 13969S Lot #3) along with the spike-in antibody (Active Motif, 61686) were utilized for quantitative ChIP-seq.

Peak calling parameters

Peaks for individual H3K27me3 replicates were called utilizing MACS2 “callpeak” function with the following parameters: - -f BEDPE -B -g hs --broad --broad-cutoff 0.2. Regions within 10kb of another were merged utilizing the Bedtools2 merge function -d 10000 (Bedtools2, version 2.26.0).

Data quality

The peak calling parameters are specified above. The qvalue cutoff for narrow/strong regions = 5.00e-02 and the qvalue cutoff for broad/weak regions = 2.00e-01.

Software

Freely available software was utilized for the ChIP-seq analysis. This includes Bowtie2 (version 2.3.2), SAMtools (version 0.1.19), Bedtools2 (version 2.26.0) and Spiker (version 1.03). MACS2 (version 2.1.1) was used for peak calling. The metaprofile plots were generated using deepTools (version 3.4.3).
